# Native architecture of a human GBP1 defense complex for cell-autonomous immunity to infection

**DOI:** 10.1126/science.abm9903

**Published:** 2024-03-01

**Authors:** Shiwei Zhu, Clinton J. Bradfield, Agnieszka Mamińska, Eui-Soon Park, Bae-Hoon Kim, Pradeep Kumar, Shuai Huang, Minjeong Kim, Yongdeng Zhang, Joerg Bewersdorf, John D. MacMicking

**Affiliations:** 1 Howard Hughes Medical Institute, Yale University School of Medicine; New Haven, CT 06510. USA; 2 Yale Systems Biology Institute; West Haven, CT 06477. USA; 3 Department of Microbial Pathogenesis, Yale University School of Medicine; New Haven, CT 06510. USA; 4 Department of Immunobiology, Yale University School of Medicine; New Haven, CT 06510. USA; 5 Department of Cell Biology, Yale University School of Medicine; New Haven, CT 06510. USA; 6 Yale Nanobiology Institute; West Haven, CT 06477. USA

## Abstract

All living organisms deploy cell-autonomous defenses to combat infection. In plants and animals, large supramolecular complexes often activate immune proteins for protection. Here, we resolved the native structure of a massive host defense complex polymerizing 30,000 guanylate-binding proteins (GBPs) over the surface of Gram-negative bacteria inside human cells. Construction of this giant nanomachine took several minutes and remained stable for hours, required GTP hydrolysis, and recruited four GBPs plus caspase-4 and Gasdermin D as a cytokine and cell death immune signaling platform. Cryo-electron tomography suggests GBP1 can adopt an extended conformation for bacterial membrane insertion to establish this platform, triggering lipopolysaccharide release that activated co-assembled caspase-4. Our “open conformer” model provides a dynamic view into how the human GBP1 defense complex mobilizes innate immunity to infection.

## One-Sentence Summary

Thousands of GBP1 proteins coat bacteria inside human cells to create an antimicrobial signaling platform.

In biological systems, environmental cues are often sensed by ligand-induced allosteric changes in cell surface receptors that rapidly transmit signals to the interior for mobilizing the desired physiological response. Within the cell-autonomous defense systems of plants and animals (1, 2), additional modalities are used to decode the outside world including intracellular protein assemblies formed via helical symmetry (3,4). These higher-order assemblies amplify innate immune signals through protein polymerization or “prionization” events to facilitate proximity-induced autoactivation of latent zymogens, caspases, and kinases. The benefits to such repetitive design are manifold: lowered signaling thresholds, all-or-none responsivity, and stable signalosome platforms that can recruit and accommodate numerous protein partners (3).

The ability to generate extended, filamentous signaling platforms stems in part from the modularity of the proteins involved. Signalosome proteins often harbor leucine-rich repeat (LRR) domains, caspase-activation and recruitment domains (CARDs), or death effector domains (DEDs) that concentrate receptors, adaptors and effectors through co-operativity (3). As a result, they yield some of the most iconic and visible structures inside immune-activated cells. These include RIG-I filaments and MAVs prion-like structures that control RNA sensing; ASC, NLRP3 and NLRC4 filaments as part of the inflammasome machinery; Myddosomes orchestrating NF-κB and IRF signaling; and NLR ZAR pentamers that underpin the plant resistosome (3-6). Collectively these large polymeric structures represent an increasingly pervasive paradigm for cell-autonomous innate immunity throughout metazoan evolution. Their mode of assembly differs from classical antigen or immunoglobulin signaling at the plasma membrane that are typically transmitted via clustered synapses (3).

To this list can now be added members of a dynamin-like GTPase family termed guanylate-binding proteins (GBPs) which undergo polymerization for generating innate immune signaling platforms and coordinating local antimicrobial activity. GBPs assemble into large multimeric structures inside *Arabidopsis*, zebrafish, mouse, and human cells, in some cases using phase-separation to further concentrate homotypic complexes (7-12). In plants, the 69-122 kDa GBP-like proteins (GBPLs) respond to inducible immune signals including salicylic acid and pipecolic acid to assemble large nuclear RNA polymerase II hubs that transcribe host defense genes during infection (7, 8). In animals, immune cytokines such as interferons (IFNs) induce 65-73 kDa GBP expression to control microbicidal or inflammasome responses within the cytosol of both immune and non-immune cells (9-13); these activities often coincide with the relocation of GBPs to the site of microbial replication where they completely “coat” targeted pathogens to build mesoscale signaling or killing platforms (9,12-16). GBP-coated pathogens can range in size from ~750 nm in diameter for *Salmonella enterica* serovar Typhimurium (*Stm*) to >5 μm for *Toxoplasma gondii* tachyzoites (17).

Assembling such a large coat complex must enlist biochemical properties synonymous with dynamin-like proteins (DLPs). DLPs typically exhibit robust GTPase activity (*k_cat_* ~2-100 min^−1^), low μM substrate affinity and nucleotide-dependent self-assembly to generate > 0.5-1 MDa complexes within cells (18,19). Hence they are “large” GTPases which often function as mechanoenzymes to deform or tubulate membranes during vesicular trafficking, organelle division or cytokinesis (18,19). Human GBPs also exhibit high intrinsic catalytic activity (*k_cat_*, ~80.min^−1^) to produce GDP plus GMP from GTP in a two-step hydrolysis reaction (20, 21). GTP hydrolysis likely initiates conformational changes leading to co-operative self-assembly of GBP dimers. Structurally, all human GBPs possess a large globular (LG) N-terminal catalytic domain and extended α-helical C-terminal tail (19); the latter spans a middle domain (MD) comprising helices α7-α11 and GTPase effector domain (GED) encompassing the final α12-α13 helices (20, 22-23). In human GBP1 and GBP5 crystal structures, the GED folds back tightly onto the LG and MD regions (20, 22-23). This could represent a closed, auto-inhibited state since substrate binding results in different geometries for isolated GBP1 when viewed by conventional electron microscopy (24). Indeed, substrate catalysis could theoretically release the GED to yield an open, active dimer which undergoes further multimerization, although high-resolution GBP structures captured directly on the pathogen surface after GTP hydrolysis have yet to be reported. In addition, GBP1, GBP2, and GBP5 each possess C-terminal CaaX motifs for isoprenyl modification to facilitate membrane binding (19, 24). Isoprenylation could offer not only anchorage but also serve as a nucleating template to deposit more GBPs on the microbial surface to build a signaling platform during innate immunity (12-16).

In the current study, we characterize a massive immune defense complex comprising nearly 30,000 GBP1 molecules assembled on the bacterial surface using cryo-electron tomography (cryo-ET). Remarkably, despite their central role for host defense across plant and animal kingdoms (25-27), the ultrastructural organization of these mesoscale GBP coat complexes on an intact pathogen membrane is currently unknown. Visualizing such structures below the light diffraction limit would enhance our understanding of how eukaryotic cells recognize and combat infection. It also yields information on GBP1 coat assembly under native conditions which trigger innate immune signaling. Each have important implications for anti-infective therapy as well as the basic biology of immune recognition and host defense within the human population.

## Human GBP1 coat size, kinetics, and stability *in cellulo*

We first examined the human GBP1 coat complex surrounding cytosolic bacteria using 4Pi single-molecule switching (4Pi-SMS) nanoscopy and fast, live three-dimensional structured illumination microscopy (3D-SIM) via an OMX-SR Blaze imaging instrument equipped with high-speed galvanometers. 4Pi-SMS is a dual objective single-molecule localization super-resolution microscope resolving 3D structures to ~20 nm (200 Å) isotropically throughout entire mammalian cells (28, 29); it enabled us to detect single GBP molecules on the surface of virulent *Stm* inside human cells. 3D-SIM imaging via the OMX-SR instrument is capable of ~180 frames.sec^−1^, ensuring coat complex assembly could be followed throughout the entire bacterial encapsulation process.

We tracked GBP1 as the forerunner of this 7-member DLP family in humans. Recent work discovered human GBP1 recruitment onto the outer membrane of cytosol-invasive pathogens including *Stm* and *Shigella flexneri* enables bactericidal activity by apolipoprotein L3 (APOL3) in IFN-γ-activated primary human intestinal epithelia, myofibroblasts and endothelium, as well as in HeLa CCL2 cells (13). We and others also reported that GBP1 recruits additional GBP family members plus endogenous human caspase-4 to stimulate cytokine release (interleukin-18; IL-18) and pyroptosis in primary intestinal human organoids, human macrophages, and human epithelial cell lines (12, 14-16). The GBP coat complex has thus emerged as a central hub for intracellular host defense and innate immune signaling in humans.

**Fig. 1.**
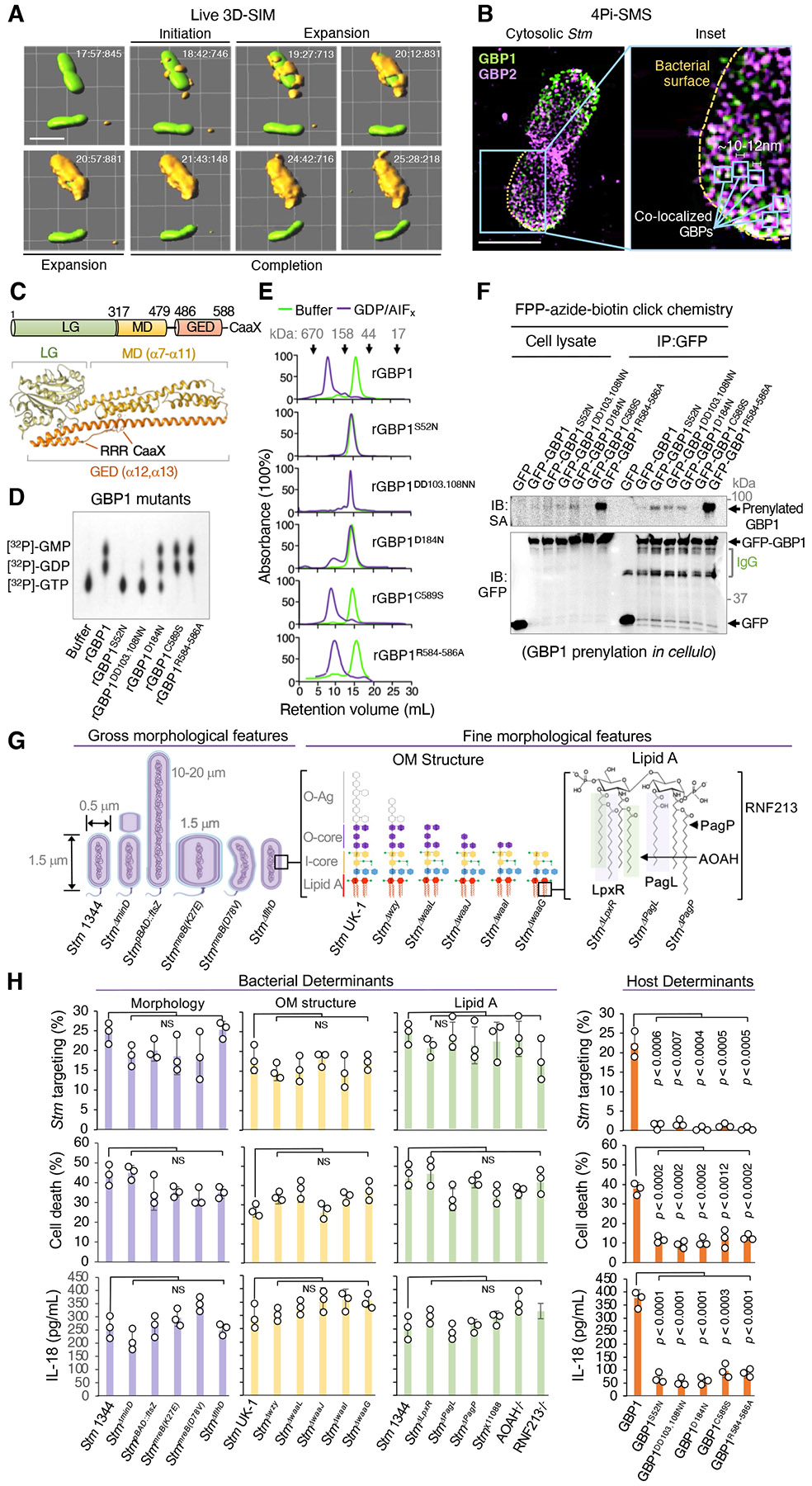
Human GBP1 coat kinetics and functional determinants *in cellulo*. (**A**) Live 3D-SIM showing full encapsulation of EGFP-expressing *Stm* by RFP-GBP1 in IFN-γ-activated HeLa cells. GBP1 coat complex initiation, expansion and completion are noted. Volume rendering via Imaris software. Maximum intensity projection of 1 of 6 similar 3D-SIM videos shown. Scale bar, 2 μm. (**B**) 4Pi-SMS nanoscopy of cytosolic *Stm* coated by endogenous human GBP1 and GBP2 detected using rat anti-GBP1 and mouse anti-GBP2 antibodies, respectively, at 2 h post-infection. Maximum intensity projection of 1 of 8 similar 4Pi-SMS images shown. Scale bar, 1 μm. (**C**) Domain structure of farnesylated human GBP1 (PBD 6K1Z) depicting catalytic large G-domain (LD), middle domain (MD) and GTPase effector domain (GED). The polybasic patch and farnesylation (CaaX) motif in the C-13 α-helix are denoted. (**D**) Thin-layer chromatography of ^32^[P]-GTP hydrolysis products for recombinant GBP1 and its mutants. Representative of 4 independent experiments. (**E**) Size exclusion chromatography profiles depicting GBP1 and its mutants in the absence or presence of the transition state analogue, GDP plus aluminum fluoride (AIF_4_^−^). Representative of 3 independent experiments. (**F**) Prenylation profile of EGFP-GBP1 and its mutants in HEK-293E cells detected using farnesylpyrophosphate (FPP)-azide-biotin click chemistry coupled to anti-GFP immunoprecipitation. SA, streptavidin-HRP. IgG, immunoglobulin heavy chains. 1 of 3 independent biological experiments. (**G**) *Salmonella* and GBP1 mutants used to examine determinants of coat complex function. Acyl chains removed via each mutation depicted by colored brackets. χ11088, *Stm*^*ΔlpxRΔPagLΔPagP*^ triple mutant. (**H**) Bacterial and host determinants in human GBP1 coat complex formation on *Stm* coating and downstream IL-18 release or cell death in IFN-γ-activated wild-type or mutant HeLa cells infected with different bacterial strains. Mean ± standard deviations for triplicates. Significant one-way ANOVA values with Bonferroni *post-hoc* test shown. NS. Not significant. Representative of 3-5 independent experiments.

To visualize real-time GBP1 coat complex formation by live 3D-SIM imaging we deleted endogenous GBP1 in human HeLa CCL2 cells via CRISPR-Cas9 and replaced it with a functional mRFP-GBP1 reporter (GBP1^−^/^−mRFP-GBP1^) detected at physiological levels to avoid over-expression artefacts (fig. S1A). When infected with fluorescent *Stm*^*EGFP*^, mRFP-GBP1 completely encapsulated individual bacilli over a ~1-6 minute time-period (Fig. 1A and movies S1, S2). Comprehensive coating was also observed in single-molecule 4Pi-SMS imaging of endogenous GBP1 and GBP2 on the bacterial surface in IFN-γ-activated GBP1^+^/^+^ cells, in some cases depicting co-localization (Fig. 1B and movie S3). Volumetric and kinetic measurements of GBP1 yielded 29,542 ± 5,156 GBP1 molecules per bacterium assembled at a rate of 103 ± 11.6 molecules.sec^−1^ (fig. S1B,C).

Such rapid kinetics required massive GBP1 co-operativity involving sequential hydrolysis of GTP and GDP for nucleotide-dependent self-assembly of GBP1 dimers on the bacterial surface as revealed by loss-of-function mutants. GTPase (GBP1^S52N^), GDPase (GBP1^DD103,108NN^) or dimerization (GBP1^D184N^) mutants blocked coat formation and downstream IL-18 release plus pyroptotic cell death (as LDH release) in stably reconstituted GBP1^−^/^−^ cells (10) (Fig. 1C to H). Notably, these N-terminal mutants were still post-translationally prenylated at a C-terminal CaaX motif (Fig. 1F and fig. S2A,B) which may otherwise help anchor GBP1 to the bacterial outer membrane (OM). Indeed, mutating the CaaX box (GBP1^C589S^) prevented both C15 farnesylation and coat attachment inside human cells (Fig. 1F to H). It did not, however, interfere with nucleotide-dependent dimer self-assembly as shown via size exclusion chromatography using a transition state analogue, GDP plus aluminium fluoride (AIF_3_^−^), in recombinant protein assays (Fig. 1E). Thus, GBP1 mutants uncoupled OM attachment from subsequent polymerization, revealing distinct steps in coat complex formation during immunity to Gram-negative infection.

OM anchorage also required a polybasic patch (amino acids 584-586) resembling lipid-binding motifs in small H-Ras GTPases (30) within the GBP1 C-terminal α-13 helix (20; fig. S2A,B). Alanine-scanning mutagenesis of all three arginines (GBP1^R584-586A^; 31) ablated coat formation and impaired downstream cytokine plus cell death signaling (Fig. 1H and fig. S2C,D). Because GBP1^R584-586A^ was heavily farnesylated inside human cells the loss of coat complex assembly was not due to R584-586A substitution interfering with lipidation of the nearby CaaX motif (Fig. 1F). Instead, it appears GBP1 farnesylation is necessary but not sufficient for OM anchorage, requiring a second site to stably engage the OM and help retain it on the bacterial surface.

The bivalent nature of this C-terminal anchor was reinforced in lipopolysaccharide (LPS)-binding profiles for the GBP1^R584-586A^ and GBP1^C589S^ mutants purified from human embryonic kidney (HEK) cells lacking endogenous GBP expression to ensure correct farnesyl linkage and post-prenyl processing (32) (fig. S3A,B). LPS is the major constituent of Gram-negative OMs (~75% in *Stm*; 33) and recently reported to bind human GBP1 (15, 16). We found *Stm* LPS captured farnesylated FLAG-tagged GBP1 at physiological pH and temperature in fluorescence anisotropy assays (*K_d_*, ~3.971 μM), whereas it failed to capture non-farnesylated FLAG-GBP1^C589S^, non-dimerized or catalytic mutants, or farnesylated FLAG-GBP1^R584-586A^ (fig. S3B). GBP1 assembly together with the farnesyl moiety therefore appears critical for *Stm* LPS engagement with the arginine patch strengthening these interactions electrostatically to maintain a stable coat (15, 16). This stability was in some cases extraordinarily long-lived: live imaging revealed a single GBP1 coat can persist for up to 2-3 hours within the human cytosol (fig. S3C). Thus, thousands of GBP1 molecules generate a highly durable signaling platform once anchored through initial farnesyl and polybasic contacts to the bacterial OM.

## Host and bacterial determinants of GBP1 coat complex assembly

Coat construction required GBP1 C-terminal attachment and N-terminal catalytic activities to polymerize over the entire *Salmonella* surface. Whether microbial activities or physical features of bacteria also influence this process was examined by engineering 13 *Stm* strains differing in size, shape, motility, and OM composition. *Stm* isolates that were longer (*Stm*^*pBAD-ftsZ*^, up to 20 μm), wider (*Stm*^*MreB K27E*^ mutants, ~2 μm diameter) or smaller (*Stm*^*ΔminD*^ minicells 250-300 nm diameter arising from aberrant septation) still recruited endogenous GBP1 in IFN-γ-primed cells to activate IL-18 release and pyroptotic cell death (Fig. 1G,H). Bent *Stm*^*MreB D78V*^ mutants likewise mobilized this pathway (34,35) (Fig. 1G,H). Hence microbial cell size, division or curvature did not seem to influence GBP1 coat formation to generate an innate immune signaling platform. Flagellin-expressing (36) and flagellin-deficient *Stm* (*Stm*^*ΔflhD*^) were both targeted by GBP1, ruling out motility or bacterial immobilization as a cue to begin coat complex assembly.

We therefore turned to the OM itself. Gram-negative bacteria harbor long polysaccharide LPS chains forming a divalent cation-crosslinked barrier that is impermeable to hydrophobic solutes (37). The LPS moiety consists of O-antigen polysaccharide, outer core galactose- and inner core heptose- and Kdo-enriched saccharides, and a lipid A module with multiple acyl chains embedded at the base by electrostatic and hydrophobic interactions (Fig. 1G). Isogenic *Stm* mutants with progressively shorter LPS chains (generated by inactivating enzymes at successive steps of the LPS biosynthetic pathway; 13,38) revealed OM truncations in *Stm*^*Δwzy*^, *Stm*^*ΔwaaL*^, *Stm*^*ΔwaaJ*^, *Stm*^*ΔwaaI*^ or *Stm*^*ΔwaaG*^ did not block GBP1 coat formation and downstream innate immune signaling (Fig. 1G,H).

Beneath these truncations we modified the lipid A module positioned at the base of the OM where it interacts with the phospholipid inner leaflet; lipid A is recognized and directly bound by caspase-4 (39). Mutations in *Stm* LpxR or PagL (that remove a 3′-acyloxyacyl moiety or single *R*-3-hydroxymyristate chain, respectively), or PagP (that palmitoylates the hydroxymyristate chain; 40) failed to prevent GBP1 coating and caspase-4 activation (Fig. 1G, H). CRISPR-Cas9 deletion of human acyloxyacyl hydrolase (AOAH^−^/^−^), which is expressed at low levels in HeLa CCL2 cells and removes secondary acyl chains from lipid A as a deactivation mechanism (41), or human E3 ubiquitin ligase ring finger protein 213 (RNF213^−^/^−^), which modifies lipid A via ubiquitinyation (42), also had no effect on coat-dependent signaling (Fig. 1G, H). Thus, functional coat formation on *Salmonella* was primarily governed by host GBP1 activities rather than lipid A modifications or other microbial determinants *in cellulo*. Human GBP1 still targeted cytosolic *Stm* irrespective of bacterial size, shape, motility, or OM composition; the latter spanned LPS chains of different length, charge, and chemical structure. Such broad ligand promiscuity may help GBP1 combat Gram-negative pathogens that modify their LPS moiety in an attempt to evade innate immune recognition and antimicrobial killing.

## Human GBP1 coat initiates a multiprotein platform for bacterial recognition

Broad multivalent GBP1 interactions with the bacterial surface provide a stable platform to recruit downstream protein partners for innate immune signaling (12,13,16). Previous work from us together with other groups found GBPs2-4 and caspase-4 form part of this GBP1 signaling platform in primary human intestinal organoids and cervical epithelial cell lines (12, 16). Here stable CRISPR-Cas9 deletions corroborated their importance with endogenous caspase-4 auto-proteolysis (denoted by the active p30 subunit), IL-18 release and pyroptotic cell death (as LDH release) significantly diminished in IFN-γ-activated GBP1^−^/^−^ and GBP2^−^/^−^ single knockout as well GBP1^−^/^−^2^−^/^−^ double knockout cells (fig. S4A to C). Single deletions of GBP3^−^/^−^ and GBP4^−^/^−^ had a lesser effect, however, *en bloc* removal of the entire 335 kb human *GBP1-7* cluster via genome engineering on chromosome 1q22.2 (GBP^Δ1q22.2^) yielded almost complete loss of downstream signaling, underscoring concerted GBP action and phenocopying CASP4^−^/^−^ and GSDMD^−^/^−^ cells (fig. S4A to C).

**Fig. 2.**
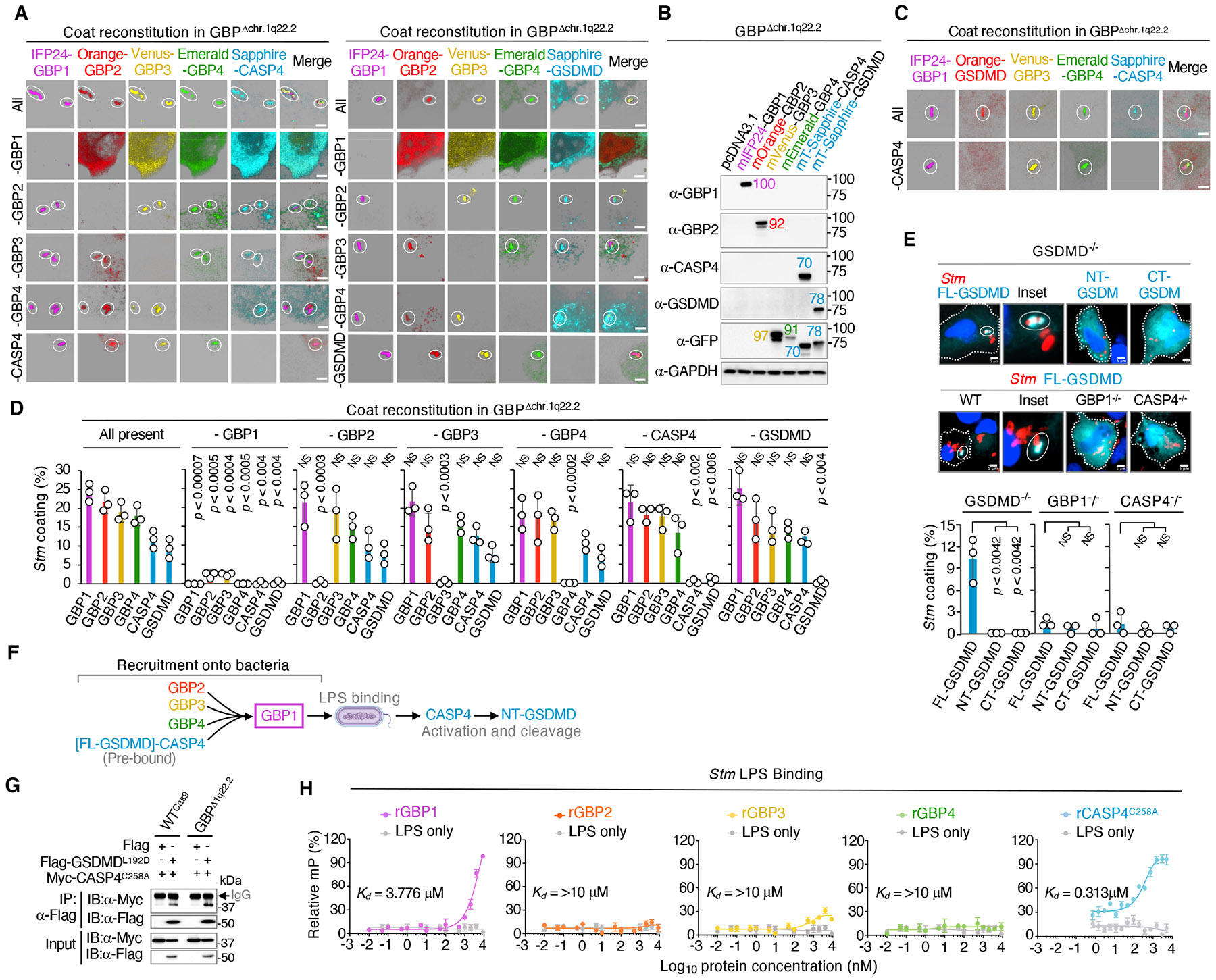
Human GBP1 initiates a multiprotein platform for cytosolic LPS recognition. (**A**) Multicolor confocal imaging of the reconstituted coat complex (GBP-COAT_450-708_) in GBP^Δ1q22.2^ mutant cells. Stepwise omission of each coat component revealed GBP1 dependence. Circles depict *Stm* targeting. IFN-γ added to ensure sufficient Caspase-4 expressed endogenously when using mT-Sapphire-GSDMD (right panel). Micrographs representative of at least 3 independent experiments. (**B**) Immunoblot of GBP1, GBP2, Caspase-4 and GSDMD by specific antibodies and anti-GFP used to detect related fluorescent proteins fused to GBP3, GBP4, Caspase-4 or GSDMD in reconstituted GBP^Δ1q22.2^ cells. (**C**) Caspase-4-dependent GSDMD recruitment shown by exchanging mOrange-GBP2 with mOrange-GSDMD since GBP2 is dispensable for reconstituted coat formation in non-activated GBP^Δ1q22.2^ cells. Scale bar, 2 μm. 1 of 3 independent experiments shown. (**D**) Quantitation of *Stm* targeted by each coat protein when all are present or following individual omission in reconstitution assays. Sapphire-GSDMD used throughout except when coexpressed with CASP4, where it was used as in panel (**A**). *n* = 156-208 events for each group from 4-5 independent experiments. NS, not significant. (**E**) Widefield imaging of full-length (FL), N-terminal or C-terminal fragments of human GSDMD fused to GFP reintroduced into GSDMD^−^/^−^ cells activated with IFN-γ and infected with *Stm* for 2 h. The GFP channel been pseudocolored turquoise and targeting of FL-GSDMD to bacteria depicted by dashed circle. Nuclear staining with DAPI. Scale bar, 5 μm. Loss of full-length GSDMD targeting in GBP1^−^/^−^ and CASP4^−^/^−^ cells below. Quantitation of GSDMD targeting in IFN-γ-treated knockout cell lines. *n* = 74-124 events for each group. NS, not significant. Micrographs and quantitation from at least 3 independent experiments. (**F**) Two-step hierarchical model showing GBP1 dependency and recruitment of full-length GSDMD by Caspase-4. (**G**) Co-immunoprecipitation of Flag-GSDMD^L192D^ by Myc-CASP4^C258A^ (to reduce cytotoxicity; 39) in wild-type and GBP^Δ1q22.2^ mutant HeLa cells. IgG, immunoglobulin heavy chain. 1 of 2 similar experiments shown. (**H**) Binding curves for recombinant coat proteins to *Salmonella* LPS-Alexa Fluor 488 in fluorescence anisotropy assays under physiological pH and temperature. Y-axis, % of maximal polarization (mP). Mean ± SD determined in triplicate for each protein concentration. Representative of 3 independent experiment experiments. Significant one-way ANOVA values with Bonferroni *post-hoc* test shown in (**D**) and (**E**). NS, not significant.

Complete *GBP* gene cluster deletion in the septuple GBP^Δ1q22.2^ mutant provided a unique tool to reconstitute the entire coat complex on an empty background and test if GBP1 is the critical organizer of this hierarchical complex on the same bacilli *in situ* (fig. S4D). Here we included full-length GSDMD as a natural caspase-4 substrate with potential bactericidal activities (43) to see if it is brought into this new supramolecular platform as well. Each coat component was fused to 1 of 7 fluorescent proteins (mAzurite, mSapphire, pmTurqouise2, pmEmerald, pmVenus, pmOrange, pmCardinal, pmIFP24; E_x_/E_m_ range, 384/450-684/708 nm) to identify compatible combinations for reconstituting GBP^Δ1q22.2^ cells, since exchange-PAINT lacked appropriate antibodies for detection of endogenous proteins (fig. S4D,E). Our color-coded orthogonal matrix (GBP-COAT_450-708_) successfully resolved 5-color objects to yield a complete signaling platform with GBPs1-4 plus caspase-4 or GSDMD all coating the same bacilli after 90-120 minutes of infection (Fig. 2A to C and fig. S4F,G).

Remarkably, this multicolored coat was completely lost if GBP1 was omitted, indicating GBP1 establishes the entire signaling cascade at the outset (10, 14) (Fig. 2A, D). Indeed, the GBP-COAT_450-708_ assay found human GBP1 was obligate for GBP2, GBP3, GBP4 and caspase-4 or GSDMD simultaneously sharing the same bacterial surface (Fig. 2D). Excluding caspase-4 had no effect on GBPs1-4, placing them upstream, but largely blocked full-length GSDMD targeting, positioning it downstream in this 6-member signaling cascade as part of two-step hierarchical model (Fig. 2C to F). Notably, N- or C-terminal GSDMD fragments mimicking the processed substrate failed to be recruited (Fig. 2E). Hence caspase-4 appears to bring only its full-length GSDMD substrate to this location for cleavage once the protease is activated by lipid A after recruitment by GBP1; this was also corroborated in individually deleted CASP4^−^/^−^ or GBP1^−^/^−^ cells (Fig. 2F). Tracking the other major caspase-4 substrate in this pathway, pro-IL-18, found it largely failed to be recruited onto the coat. Hence, GSDMD is a new component of the GBP1-4/CASP4 signaling complex as part of a 2-step hierarchical model (Fig. 2F). Its interaction with caspase-4 likely occurs before reaching the GBP platform as shown by co-immunoprecipitation assays in GBP^Δ1q22.2^ cells (Fig. 2G).

This hierarchical model was further supported by LPS binding profiles (Fig. 2H). FLAG-GBP1, -GBP2, -GBP3, -GBP4 or -caspase-4^C258A^ (preventing auto-proteolysis; 39) were purified from human HEK cells to avoid bacterial contaminants and ensure proteins were post-translationally modified; GBP1 (*K_d_*, ~3.776 μM) and caspase-4^C258A^ (*K_d_*, ~313 nM) strongly interacted with *Stm* LPS as major OM binding proteins (13-14, 39) (Fig. 2H). GBP1 therefore serves as the principal organizer of this multiprotein complex atop the coated bacterium *in situ* (12,16). Its assembly facilitates bacterial recognition of the LPS lipid A moiety by caspase-4 that can recruit GSDMD as a subsequent step to activate cell death and cytokine release downstream.

## GBP1 defense complex triggers bacterial LPS release *in cellulo* and in reconstituted systems

**Fig. 3.**
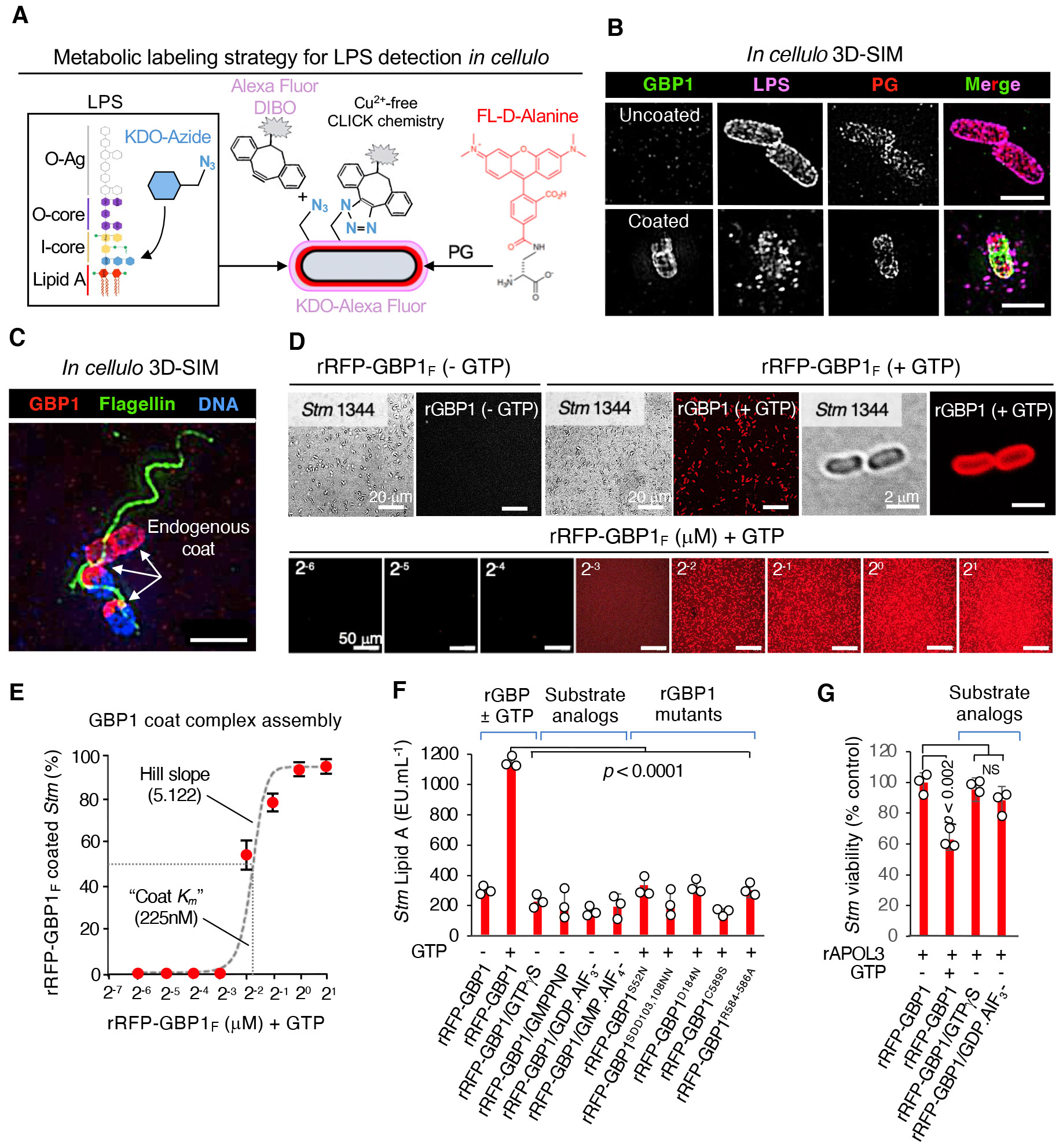
The human GBP1 coat complex triggers LPS release *in cellulo* and in cell-free systems. (**A**) Fluorescent labeling strategy for LPS using copper-free click chemistry with a dibenzocyclooctynol (DIBO) alkyne intermediate. KDO, 3-deoxy-D-manno-octulosonic acid. (**B**) 3D-SIM imaging of cytokine-activated HeLa cells (1,000U IFN-γ, 18h). Images collected at 2 h post-infection with pre-labelled *Stm* at MOI of 20. Endogenous GBP1 detected by anti-GBP1 and LPS detected with anti-Sal-O antibody for verification (pseudocolored magenta). Maximum intensity projection of 1 of 5 similar 3D-SIM images. Scale bar, 2μm. (**C**) GBP1-coated cytosolic bacteria harbor flagellin detected by anti-Fli-C antibody in 3D-SIM imaging. Maximum intensity projection of 1 of 4 similar 3D-SIM images. (**D**) (Top) Farnesylated rRFP-GBP1_F_ (2 μM) coat assembly on *Stm* ± GTP (2 mM) imaged by confocal microscopy. Representative of 10-12 micrographs shown. (Bottom) rRFP-GBP1_F_ coating across increasing diluents imaged by confocal microscopy. (**E**) Best-fit interpolation curve of means ± SD together with regression analysis revealed half maximal (“coat *K_m_*”) and Hill slope values. 1 of 3 similar experiments shown. (**F**) Soluble lipid A release by LAL in rRFP-GBP1_F_ coat reconstitution assays (triplicate ± SD) on live unfixed bacilli. Substrate analogues and GBP mutants denoted. One of 6 independent biological experiments shown. (**G**) *Stm* killing assay by rAPOL3 requires LPS disruption by the GBP1 coat complex. Significant one-way ANOVA values with Bonferroni *post-hoc* test shown for **F,G**. NS, not significant. One of 3 independent experiments shown.

How does the GBP1 coat complex promote LPS recognition by caspase-4, especially since lipid A is buried at the base of the OM? We initially probed LPS release in live bacteria *in cellulo* (Fig. 3A,B). First, copper (Cu^2+^)-free CLICK chemistry was used to label *Stm* LPS with Alexa Fluor attached via Kdo-azide derivatives adjacent to lipid A within the inner core (Fig. 3A). Here anti-*Salmonella* O-antigen antibody was used in conjunction to verify the KDO-Alexa Fluor signal which decreases during transit to the cytosol. We likewise incorporated fluorescent D-alanine into the L-Ala-D-*meso*-diaminopimelate-D-Ala-*O*-Ala pentapeptide via metabolic labelling of the underlying peptidoglycan scaffold in active bacteria (44) (Fig. 3A). 3D-SIM found complete GBP1 coating triggered release of LPS but did not seem to disturb the *Stm* peptidoglycan layer within the cytosol of IFN-γ-activated HeLa cells (Fig. 3B), confirming GBP1 can promote caspase-4 ligand availability *in cellulo*. Other exteriorized structures such as flagella were still evident on GBP1-coated bacteria (Fig. 3C); hence GBP1 primarily affected OM disruption, leaving the underlying PG scaffold and flagellar apparatus intact.

We next directly assayed lipid A release to test if GBP1 was indeed sufficient for LPS liberation. Here we used a cell-free reconstitution system to measure soluble lipid A which cannot be undertaken *in situ* due to contamination of the host cell cytosol by whole bacteria. Incubation of farnesylated recombinant RFP-GBP1 with axenic *Stm* found >95% of bacteria were fully coated within 60 minutes after addition of GTP substrate (Fig. 3D). Bacterial encapsulation by rRFP-GBP1 followed a highly accelerated sigmoidal curve yielding a half maximal value (“coat *K_m_*”) of 225nM and steep Hill slope of 5.122 (Fig. 3E). Notably, this all-or-none behavior did not arise from crossing a phase transition boundary since rRFP-GBP1 does not phase separate, unlike plant GBPLs that possess a C-terminal intrinsically disordered region to generate biomolecular condensates during infection (6,7) (fig. S5A). Pronounced coating was evident irrespective of *Stm* size or LPS status; comparison with Gram-negative *P. aeruginosa* and Gram-positive *L. monocytogenes* showed the latter two bacteria had lower levels of rRFP-GBP1 encapsulation (fig. S5B,C).

Importantly, reconstituting the GBP1 coat complex triggered *Stm* LPS release as measured by limulus amebocyte lysate (LAL) assay that detects the soluble lipid A moiety. Here unfarnesylated rRFP-GBP1^C589S^ and farnesylated catalytic or assembly mutants that could not coat *Stm* failed to release LPS even after addition of GTP, mimicking the results seen inside human cells (Fig. 3F). Omission of GTP or substitution with non-hyrolyzable GTP analogues (GTP-γ-S or GMPPNP) or transition-state mimics (GDP.AIF_3_^−^, GMP.AIF_4_^−^) also failed to trigger GBP1-dependent LPS release since these analogues cannot support hydrolysis-driven GBP1 coating on bacteria (Fig. 3F). The amount of lipid A released by GTP-dependent rGBP1 assembly on *Stm* greatly exceeded the *K_D_* range of caspase-4 (37) yet comprised < 1% of the total LPS present (based on 2 x 10^6^ molecules of LPS per *Stm*; 31). Hence the GBP1 coat complex disrupts the LPS outer leaflet; depending on the extent of this disruption, it may coincide with inflammasome activation or even bacterial killing (13,41). Indeed, co-incubating rGBP1 with human rAPOL3 led to loss of bacterial viability (Fig. 3G) because OM disruption by GBP1 allows APOL3 access to the *Stm* inner membrane underneath (13). Such dependency was confirmed using non-hydrolyzable GTP-γ-S or GDP.AIF_3_^−^ that not only prevented GBP1 coat formation and LPS release (Fig. 3F), but also subsequent rAPOL3-mediated killing (Fig. 3G). Thus, insertion of human GBP1 seems to disrupt lateral LPS-LPS interactions to compromise OM integrity. This not only activates the caspase-4 inflammasome pathway but allows the passage of small antimicrobial proteins such as APOL3 to directly kill pathogenic bacteria (13).

## A bacterial minicell system enables cryo-EM and cryo-ET of the native GBP1 coat

**Fig.4.**
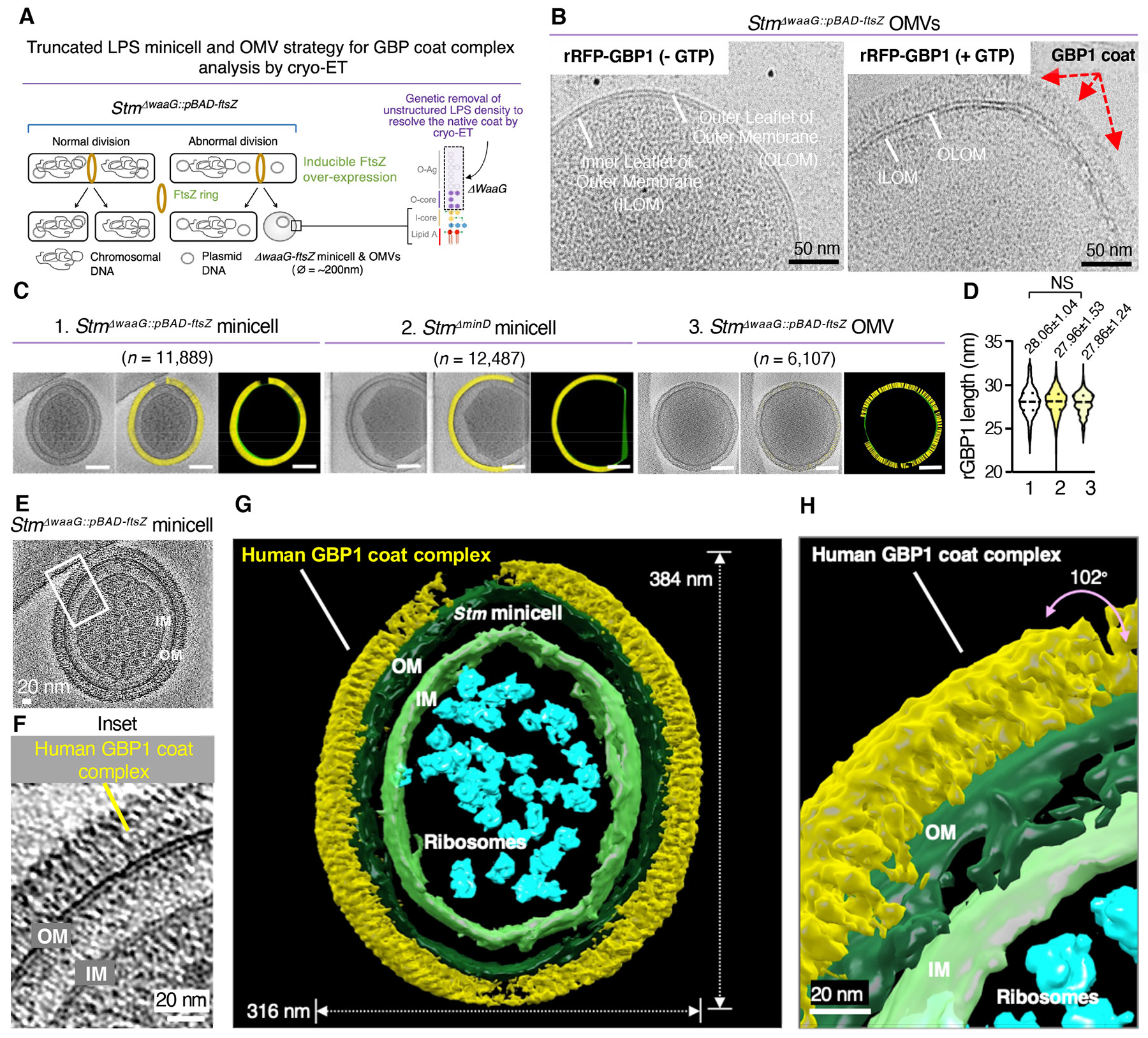
Native GBP1 coat complex on the *Salmonella* surface observed by cryo-EM and cryo-ET. **(A)** Strategy for constructing *Stm*^*ΔwaaG::pBAD-ftsZ*^ minicells and OMVs missing LPS O-antigen and outer core for reduced noise detection of the native GBP1 coat complex. **(B)** GTP-dependency of rRFP-GBPl_F_ coat formation on the *^ΔwaaG::pBAD-ftsZ^* OMV surface shown in 200 kV cryo-EM images. Black dots, 6 nm diameter fiducial beads. (**C)** Measurement of coat length from 300 kV cryo-ET images using computer script quantitation (yellow) for LPS-O antigen and outer core truncated minicell (1, *Stm*^*ΔwaaG::pBAD-ftsZ*^ ), LPS wild-type minicell (2, *Stm*^*ΔminD*^) and LPS-O antigen and outer core truncated OMV (3, *Stm*^*ΔwaaG::pBAD-ftsZ*^). Number of measurements in brackets. (**D**) Violin plots of GBP1 conformer length (nanometers, mean ± SD) for each measured sample. NS, not significant across groups using one-way ANOVA with Bonferroni *post-hoc* test from 3-4 independent experiments. (**E**) Representative tomographic slice of *Stm*^*ΔwaaG::pBAD-ftsZ*^ minicell completely coated with rRFP-GBP1_F_ after GTP hydrolysis. (**F**) Enlarged view of the boxed inset from (**E**). (**G and H**) 3D segmentation of the same rRFP-GBP1_F_ coated *Stm*^*ΔwaaG::pBAD-ftsZ*^ minicell shown in panel E and inset panel F. Tomographic series collected over 102° tilt range. OM, outer membrane. IM, inner membrane.

Our reconstituted coat complex provided us with the opportunity to view GBP1 assembly and insertion into the bacterial outer leaflet for triggering LPS disruption. Here GBP1 conformers could be delineated with powerful imaging tools such as cryo-EM and cryo-ET. We engineered bacterial minicells and outer membrane vesicles (OMVs) since they are considerably smaller than isogenic rod-shaped bacilli but retain intact features of the pathogen OM, unlike artificial liposomes or soluble LPS (Fig. 4A). More importantly, the reduced sample thickness of minicells improves resolution in cryo-ET samples (34) and negative-stain EM initially confirmed *Stm* minicells and OMVs are coated by rRFP-GBP1 like the parental *Stm* 1344 strain (fig. S6A,B).

Minicells arise from abnormal asymmetric cell division (Fig. 4A); we found over-expression of a septation gene, *ftsZ*, yielded the smallest *Stm* minicells (~150-300nm) that could still be isolated intact by differential centrifugation along with OMVs (fig. S6C). We deliberately introduced *ftsZ* over-expression into a *waaG*-deficient background lacking the LPS O-antigen and outer core segment (*Stm*^*ΔwaaG::pBAD-ftsZ*^) because these segments have unstructured density which interfere with sub-tomogram averaging (43) and removing it would allow us to see more of the native GBP1 dimer near the point of OM insertion. Both O-antigen and outer core segments are also dispensable for GBP1 coat attachment and downstream signaling inside human cells because the lipid A region is still intact (Fig. 1G,H). Our dual genetic strategy was therefore devised to limit non-specific sample noise during collection of tilt images by cryo-ET.

Cryo-EM at 200kV revealed successful reconstitution of the GBP1 coat complex on *Stm*^*ΔwaaG::pBAD-ftsZ*^ minicells and OMVs in comparative samples with rRFP-GBP1 ± GTP (Fig. 4B and fig. S6D,E). Preliminary EM measurements found GBP1 spanned ~25-27nm tightly juxtaposed over the entire bacterial surface (fig. S6E). In a recent crystal structure of farnesylated human GBP1 bound to the non-hydrolyzable GTP analogue, GMPPNP (PDB 6K1Z), the protein is half this length (12.89 nm; 22) with the final α13 helix folded back onto the α12 C-terminal segment in a “closed” conformation (fig. S6EF). To span the ~25-27 nm measured via cryo-EM at least 2 “closed” GBP1 molecules vertically positioned on top of one another would be needed, or 4-6 molecules arrayed horizontally and perpendicular to the outer leaflet (44). Alternatively, AlphaFold2 modeling (45) found GBP1 may undergo dynamic C-terminal extension of its GED to present a fully unhinged C15 farnesyl group to the OM (fig. S6F). This “open” extended conformer had a predicted length of 278 Å which also fits the EM measurements. Each potential configuration was probed by cryo-ET along with biochemical evidence to discern how GBP1 directly operates on the bacterial surface under native conditions in the presence of its *bone fide* substrate, GTP.

## Cryo-ET reveals thousands of GBP1 conformers insert into the bacterial OM

**Fig. 5.**
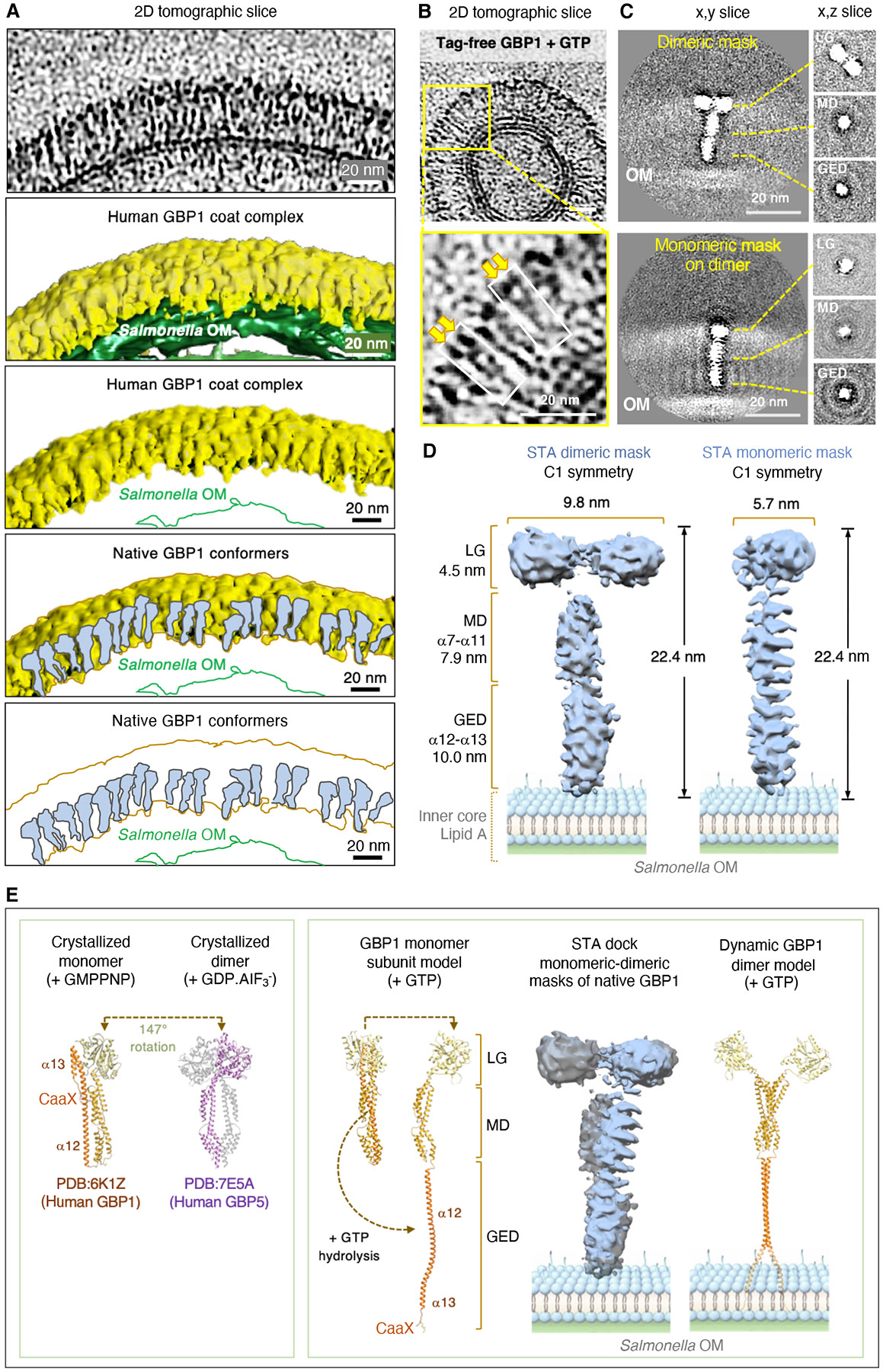
Human GBP1 could adopt a dynamically “open” conformer when assembled on the bacterial OM following GTP hydrolysis. **(A)** 3D segmentation of the human GBP1 coat complex with multiple upright GBP1 conformers attached to the bacterial outer membrane (OM) shown at ~30 Angstroms by cryo-ET in the presence of 2 mM GTP. A 2D tomographic slice is shown at the top. (**B**) Representative 2D tomographic slice of *Stm*^*ΔwaaG::pBAD-ftsZ*^ OMVs coated with tag-free GBP1 in the presence of GTP. Yellow box highlights elongated GBP1 conformers containing dimers (yellow arrows) within the inset. Scale bar, 20 nm. (**C**) (Top) Asymmetric GBP1 dimer on the bacterial OM captured via a larger mask. Image shows the 182^nd^ slice of 256 slices used for generating a 3D volume of the GBP1 dimer sub-tomogram average (STA) within 256*256*256 voxels. Tomogram rotated counterclockwise at 45° to reveal both large GTPase domains (LGs) of the dimer. Right panels are cross-sections (x,z-slices) of the GBP1 STA at the LG, MD, and C-terminal GED in the original orientation. (Bottom) Smaller mask on one monomeric subunit of the native GBP1 dimer which yielded higher resolution. Image shows the 155^th^ slice of 256 slices used for generating a 3D volume of the GBP1 STA within 256*256*256 voxels. (**D**) STA of native human GBP1 directly on the bacterial outer membrane. Native dimer (17 Angstrom final resolution) and monomeric subunit of the dimer (9.7 Angstrom final resolution) show α12 and α13 helical domains extending down into the bacteria outer membrane. (**E**) (Left) Monomer and dimer of models of crystallographic GBP structures in the presence of substrate analogues. (Right) Computational GBP1 monomer subunit and dimer models incorporating a tilted LG domain of the GBP5 dimer (PDB:7E5A) together with an extended GED on the bacterial membrane following hydrolysis of its natural substrate, GTP. Monomeric STA docked onto its dimeric counterpart from cryo-ET studies is placed in-between for comparison. The positions of LG, MD and GED are denoted, along with the CaaX motif for C15-farnesyl attachment.

We next used 300 kV cryo-TEM to acquire high contrast 3D images in dose-fractionated mode of a human GBP1 coat complex fully assembled on the bacterial surface (Fig. 4C to H and fig. S7A to L). rRFP-GBP1 fluorescence initially helped us to locate coated bacilli within vitrified samples while the position of RFP did not alter the overall length of GBP1 (fig. S6F). Mean conformer lengths of ~280 Å were found across 30,483 measurements of *Stm*^*ΔwaaG::pBAD-ftsZ*^ and *Stm*^*ΔminD*^ minicells plus OMVs (Fig. 4C,D; movies S4 and S5). Here elongated GBP1 conformers radiating from the bacterial surface was discernible within multiple tomographs (Fig. 4E to H and fig. S7G). 44,891 particles collected during coarse classification yielded 15,683 particles for 3D segmentation of this native host defense complex (Fig. 4G,H). It resolved a massive coat fully surrounding *Stm*^*ΔwaaG::pBAD-ftsZ*^ minicells up to 384 nm in diameter (movie S6). Zoomed-in views revealed what appear to be vertically upright GBP1 conformers often aligned in register when attached to the OM (Fig. 5A).

To verify individual protein configurations within this native coat, we next enlisted tagless rGBP1 to ensure RFP was not interfering with lateral packing and applied tighter final masks on 12,858 particles for subtomogram averaging (STA)(Fig. 5B and fig. S8A to C). Such refinement necessitated user-side scripting to accommodate the small 68 kDa size of untagged GBP1, its flexibility, and dense array on the bacterial OM. Using this strategy, we were able to resolve the larger GBP1 dimer and its smaller monomeric subunit to ~9-17 Å directly on the bacterial surface (Fig. 5C, D and fig. S9A to D). At these resolutions both the LG and α-helical MD plus GED regions could be delineated (Fig. 5D and fig. S9A,C). Native GBP1 adopts an asymmetric dimer with the LGs twisted and tilted relative to each other when attached to the bacterial OM (Fig. 5D). This may resemble the tilted LGs found in the crystallized dimer of human GBP5 (PDB:7E5A; 23) although the α12 and α13 GED helices appear extended into the LPS inner core and lipid A regions for native GBP1, yielding a minimum length of 22.4 nm (Fig. 5D, E). Hence, one possibility arising from our cryo-ET analysis is that human GBP1 operates as an “open” conformer in its functional state (Fig. 5E); this contrasts with all monomer or dimer GBP crystal structures reported so far which position the GED in a “closed” conformation (Fig. 5E).

Enumerating these native conformers and the bacterial surface area present within each subtomogram segment, along with overall size measurements of *Stm*^*ΔwaaG::pBAD-ftsZ*^ minicells, found 11,760 ± 735 GBP1 proteins assembled on the OM, yielding a >1GDa structure. For a mature rod-shaped *Stm* this extrapolates to ~22,000-32,000 GBP1 molecules per bacterium, consistent with earlier light microscopy estimates *in situ*. Cryo-ET thus provided a new structural view of this giant immune complex where thousands of GBP1 dimers may stretch their C-terminal GED domain to ~270-280 Å for anchoring the farnesyl tail and insertion of the nearby polybasic patch.

This mesoscale coat also appeared evident *in situ*. Focus ion beam-(FIB)-milled lamella together with correlative light and electron microscopy (CLEM) found GBP1-coated bacteria inside human cells (fig. S10A to D). Here longer *Stm*^*pBAD-ftsZ*^ bacilli were needed to detect rare coating events in thin ~150-300 nm FIB-milled lamella while EGFP-GBP1 expression in a GBP^Δ1q22.2^ background ensured it was the only GBP family member targeting bacilli (fig. S10A). This dual strategy succeeded in resolving what appear to be GBP1 coat complexes on CLEM-validated bacteria that had escaped into the cytosol; vacuolar *Stm* were devoid of EGFP-GBP1 as a negative control (fig. S10B to D and fig. S11A,B). Individual GBP1 dimers could not be delineated at this resolution, however, the ~30 nm EGFP-GBP1 boundary surrounding *Stm* closely resembled the length of reconstituted coats in cell-free assays (fig. S11A). Hence the conformational changes seen *in vitro* probably operate *in cellulo* to generate this massive GBP1 defense complex.

## A conformationally dynamic model for GBP1 defense complex assembly

**Fig. 6.**
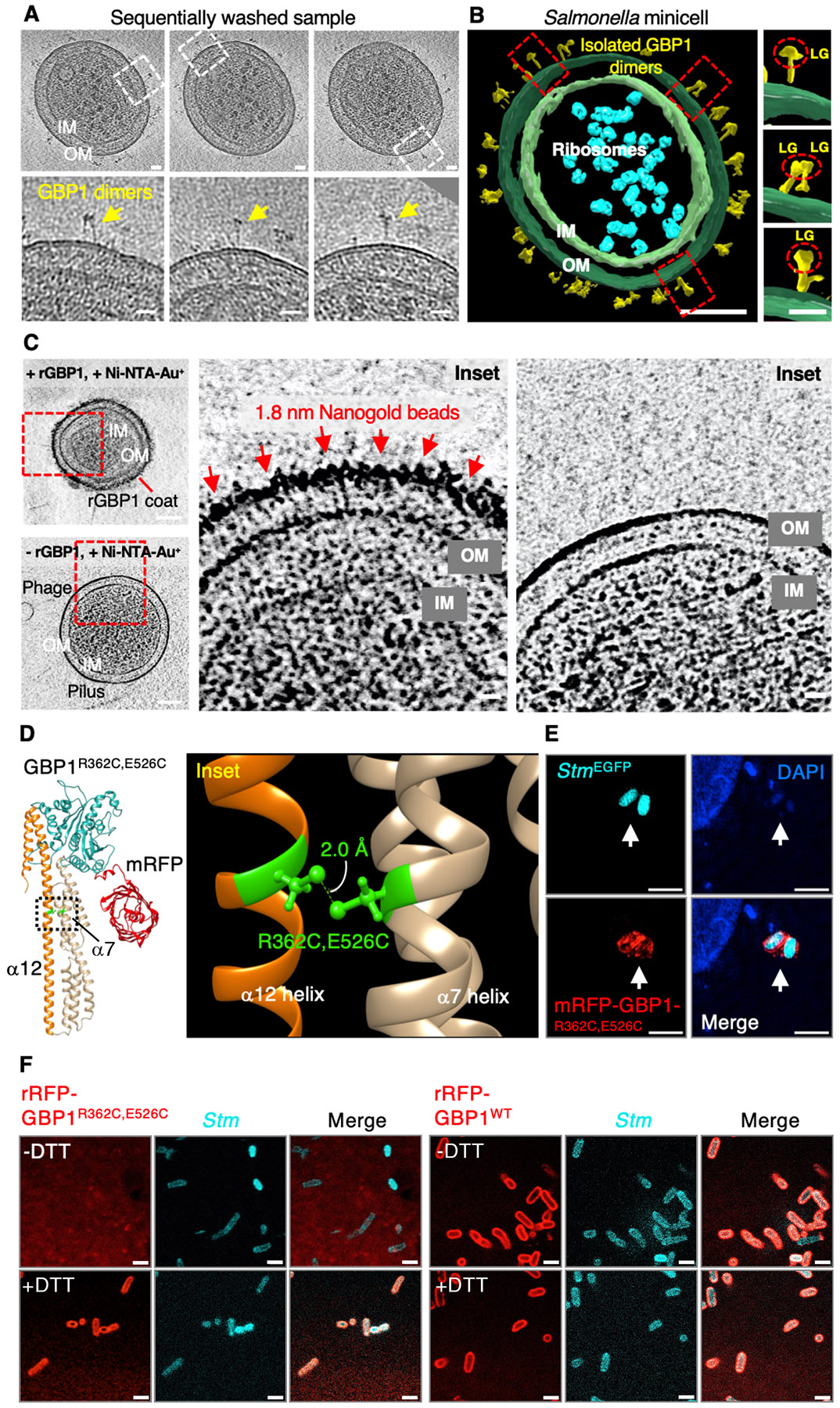
Validation of the dynamically “open” conformer model for the GBP1 coat complex. **(A)** Direct visualization of isolated GBP1 dimers on *Stm*^*ΔMinD*^ minicell after GTP hydrolysis followed by the sequential wash protocol to remove crowdedness. Inset, a zoom-in view showing isolated GBP1 conformers (yellow arrows). **(B)** 3D segmentation of the *Stm*^*ΔMinD*^ minicell after wash treatment. Inset, zoom-in view of isolated GBP1 depicting the large globular GTPase domain (LG; dashed circles) at the periphery with helical stalk underneath. **(C)** Topological evidence of the His_6_-GBP1 upright conformer. Representative tomographic slice of *Stm*^*ΔMinD*^ minicell with and without His_6_-GBP1 coat complex in the presence of GTP. Inset is the zoom-in view at right. His_6_-GBP1 GD labeled at the outer perimeter of the coat with 1.8 nm Ni-NTA-nanogold particles are shown in red arrow. OM, outer membrane, IM, inner membrane. Bars in left panel (100 nm) and in the zoom-in view (20 nm), respectively. One of at 3 independent experiments. Tomographs denoised with cryoCARE software to delineate nanogold particles. **(D)** Cross-link design between α7 in the MD and α12 in the C-terminal GTPase effector domain. Inset, Residues selected for cysteines substitution for forming a disulfide linkage. **(E)** *In cellulo* examination of mRFP-GBP1 with Cys replacements targeting onto the *Salmonella* surface indicating cysteine mutations do not grossly alter GBP1 function inside human cells. GFP-expressing bacteria targeted with RFP-GBP1^R362C-E526C^ shown by arrows. The GFP channel been pseudocolored turquoise. 1 of 2 similar experiments shown. **(F)** (Left) Release of the covalent α7-α12 crosslinked Cysteines (recombinant RFP-GBP1^R362C-E526C^) by DTT allows GTP-dependent assembly on the bacterial OM in reconstitution assays. (Right) Wild-type recombinant RFP-GBP1 is unaffected by the presence of DTT in GTP-dependent coat assays. Bar, 2 μm. 1 of 3 similar experiments shown.

The conformationally dynamic model of human GBP1 was further validated by three separate approaches. First, we devised sequential wash conditions to reduce protein crowding within the coat complex itself. It enabled us to observe isolated GBP1 dimers still attached to the surface of *Stm* OMVs and minicells *in vitro* (Fig. 6A,B and fig. S12A,B). Tomographic images of 66 isolated GBP1 proteins found all had the globular LG positioned at the perimeter with the extended C-terminus anchored underneath at the base; these isolated dimers spanned 25.08-27.44 nm, suggesting an extended conformation (Fig. 5D-F and fig. S12C,D). Second, nanogold labeling validated this orientation. Here we chose 1.8 nm Ni-NTA-Nanogold particles to bind a histidine tag (His_6_) fused to the N-terminal LG of GBP1 for detecting its location within mature coat. Labeling was observed exclusively at the outer border (Fig. 6C), consistent with the LG being positioned at the top of the coat as seen for isolated dimers (Fig. 6A,B). Importantly, the exposed bacterial OM did not bind Nanogold particles in the absence of His_6_-GBP1, indicating the labeling signal originated from the protein itself (Fig. 6C). Attempts to introduce a functional His_6_ sequence near the GBP1 C-terminus proved impractical due to the presence of the CaaX motif used for farnesylation and polybasic R584-586A sequence needed for OM anchorage. Nonetheless, cryo-ET of both washed OMVs and nanogold labeling both validated the length and the orientation of GBP1 dimer on the Gram-negative bacterial surface.

Lastly, a requirement for dynamic opening of the GBP1 GED was assessed via a cysteine replacement assay. Here seven cysteine paired mutants (I369C-E533C, E366C-G530C, I365C-H527C, R362C-E526C, I365C-G526C, G361C-S523C, G389C-K520C) were generated spanning the C-terminal GED α12 helix and its spatially adjacent α7 MD helix to enable disulfide cross-linking of the “closed” conformer (fig. S13A); this prevented the C-terminal α12,13 region from opening to ~280 Å unless exposed to a reducing agent such as DTT. To ensure the introduction of Cys-Cys pairs did not lead to gross structural alterations having effects beyond opening of the C-terminal hinge, we first tested if they could still target *Stm* within the reducing environment of the human cytosol. All seven Cys-Cys pairs coated cytosolic *Stm* in HeLa cells (fig. S13B). Of these mutants, we chose the middle R362C-E526C mutant with the shortest Cys-Cys interbond length (2 Å) to make recombinant RFP-GBP1 for direct cell-free coating assays after confirming its normal activity *in situ* (Fig. 6D,E and fig. S13A,B). In the absence of DTT, the “closed” disulfide-linked R362C-E526C conformer completely failed to coat *Stm* even when given its GTP substrate, unlike its wild-type rRFP-GBP1 control (Fig. 6F). Addition of DTT, however, fully restored bacterial encapsulation by the R362C-E526C mutant (Fig. 6F). Thus, biochemical evidence supports the requirement for dynamic opening of the human GBP1 dimer to ~280 Å for assembly on the bacterial surface. Here the C15 farnesylated tail anchors GBP1 to the OM with the catalytic LG domain positioned at the perimeter to generate this unique host defense platform.

## Discussion

Higher-order protein assemblies help amplify innate immune signaling and spatially regulate signal propagation by localizing partners at the site of ligand recognition (1,3). Such assemblies form on the host plasma membrane, mitochondria, peroxisomes, and chloroplasts (1,46). They also occur in the cytosol, nucleus, ER and endosomal network, in some cases yielding membrane-less condensates via liquid-liquid phase separation (47), as recently discovered for plant GBPLs during cell-autonomous defense against bacterial phytopathogens (7,8).

By contrast, human GBP1 builds a multiprotein complex upon a completely foreign object - the Gram-negative bacterial OM. This huge nanomachine solicits GBPs2-4, caspase-4 and full-length GSDMD as part of a 6-member platform to propagate cytokine and cell death signaling in multiple human cell types (10,16). We found human GBP1 is the central organizer of this platform and enlists nucleotide-dependent hydrolysis to self-assemble like other members of the DLP superfamily of large GTPases (18). GTP-driven GBP1 co-operativity gave a sigmoidal *Stm* coating curve on bacteria that steeply accelerated above 125 nM, resembling other “prionizing” proteins where all-or-none responsivity occurs once a concentration threshold is reached (3). Here anchorage to LPS may help accelerate GBP1 catalysis and oligomerization (16). Both GTPase and GDPase activities contributed to GBP1 responsivity via GTP/GDP turnover as part of a 2-step enzymatic process (15, 21). Because hydrolysis of GTP/GDP liberates large amounts of Gibbs free energy the GBP1 coat conforms to a nanomachine by performing “work” in establishing this massive signaling platform. It may explain why transition state analogues such as GDP.AIF_3_^−^ fail to recapitulate the native coat on intact bacteria (15) despite helping GBP1 form dimers (20,21). Instead, multiple rounds of GTP and GDP hydrolysis are needed to continually drive assembly of adjacent dimers that probably undergo lateral interactions to stabilize the coat in register. Cryo-ET suggests these lateral interactions could result from GTP-induced changes to the twisted LG itself given GBP1 dimers appeared slightly asymmetric when embedded within the native coat complex.

The functional importance of GTP hydrolysis was further reinforced by its ability to trigger GBP1-dependent LPS release which activates both caspase-4 and sensitizes bacteria to APOL3-mediated killing (13). Again, transition-state (GDP.AIF_3_^−^, GMP.AIF_4_^−^) or non-hydrolyzable analogues (GTPγS, GMPPNP/GppNHp) failed to elicit GBP1-dependent disruption of the OM to liberate LPS. These results highlight the limitation of using substrate mimics to probe GBP1 defense complex formation and functionality on the pathogen surface, despite their usefulness in earlier studies of isolated GBP1 (20-21, 24).

Cryo-ET provided us with structural information about GBP1 and its supramolecular architecture on the Gram-negative *Salmonella* surface in its native state. Previous crystal structures of GBP1 used full-length recombinant protein produced in *Escherichia coli* to capture the monomeric (apo) state ± GMPPNP (20,22). The N-terminal G-domain also produced in bacteria was crystallized as a homodimer in the presence of multiple non-hydrolyzable nucleotides (GMPPNP, GDP.AIF_3_^−^, GMP.AIF_4_^−^, GMP) (21). Both full-length human GBP1 crystal structures position α12 and α13 GED helices tucked up against the α7-α11 MD in a folded conformation, whereas under native conditions we found recombinant GBP1 produced in human cells is fully stretched with the α12 and farnesylated α13 helices inserting vertically into the bacterial outer leaflet. Identifying an “open” GBP1 conformer as the principal repetitive unit of the mature coat complex reinforces the capacity of cryo-ET to yield insights into the behavior of assembled proteins on their natural membrane targets and in the presence of their natural substrate, in this case GTP (51).

Our attempts to resolve this massive GBP1 defense complex assembled on its physiological surface proved challenging across two scales: first, the small size of GBP1 dimers (~140kDa), and second, the giant ~1.5GDa size of the final polymer. Determining the length and orientation of individual GBP1 molecules to 9.7 Å among thousands of identical proteins benefitted not only from post-acquisition masking but also from bacterial genetics plus recombinant protein preparation. Purified 150-300 nm minicells and OMVs with reduced thickness helped improve the quality of our tilt images. It was further aided by genetic removal of the O-antigen and outer core to prevent unstructured LPS density interfering with GBP1 C-terminal resolution (43). Here part of the GED of GBP1 otherwise hidden within the inner leaflet could be reconstructed from tomographs to help confirm the dynamically open model. Lastly, farnesylated GBP1 purified directly from human cells via FPLC also ensured proper OM membrane anchorage and insertion. C15 lipidation requires sequential addition by human farnesyltransferase, tripeptide removal by CAAX carboxypeptidase (RCE1), and carboxy-group methylation by isoprenylcysteine carboxymethyltransferase (ICMT) (32). Post-prenyl processing thus brings the fully modified farnesyl tail almost adjacent (3 amino acids apart) to the triple arginine patch, creating a powerful bipartite anchor. OM insertion of the polybasic motif likely undergoes electrostatic interactions with the negatively charged PO_4_^−^ groups of lipid A and inner core saccharides while farnesylation makes the tail more hydrophobic (15,16). Together these modifications enabled STA of human GBP1 bound to Gram-negative bacteria. It should help annotate tomographic densities of FIB-milled human cells now we have established initial conditions to detect GBP1-coated bacteria *in cellulo*.

Overall, our 3D reconstruction from cryo-ET elucidates the mesoscale architecture of a distinctive host defense structure - the massive GBP1 coat complex – that co-operatively functions on the surface of microbial pathogens inside the human cytosol. Our findings reinforce the importance of higher-order protein assembles within the innate immune systems of animals and plants. These nanomachines concentrate signaling and effector proteins for rapid mobilization of cell-autonomous resistance to infection.

## Materials and Methods:

### Antibodies & reagents

#### Antibodies

Antibodies used were anti-Flag M2 (F1804, Sigma), anti-HA (16B12, Biolegend), anti-Myc (9E10, ThermoFisher), anti-GFP (11814460001, Roche; A0174, Genscript), anti-GST (1E5, Origene), anti-DsRed (sc-390909, SCBT), anti-GBP1 (sc-53857, SCBT), anti-GBP2 (sc-10581, SCBT), anti-*Salmonella* O Group B antiserum (240984, BD), anti-flagellin (FliC-1, BioLegend), anti-β-actin (ab6276, Abcam), anti-GAPDH 41335; SCBT), anti-IL-18 (PM014; MBL), anti-Caspase-4 (clone 4B9; Enzo), anti-GSDMD (NBP2-33422, Novus Biologicals). See Table S1 for applications.

#### Reagents

Guanosine 5′-triphosphate sodium salt (G8877), GTP-γ-S (tetralithium salt; G8634), guanosine 5′-diphosphate sodium salt (GDP; G7127), guanosine 5′-monophosphate sodium salt (GMP; G8377), aluminum trifluoride (449628), chloramphenicol (220551), Ficoll (F5415) and biotin (B4501) (Sigma/Millipore); Guanosine-5'-[(β,γ)-imido]triphosphate (tetralithium salt)/GMPPNP/GppNHp (Jena Biosciences; NU-401-50); recombinant human IFN-γ (285-IF/CF; R & D Systems; 285-IF); *Salmonella minnesota* LPS-Alexa Fluor 488 (ThermoFisher; L23356).

### Bacterial strains

Bacterial strains were generated in-house or kindly provided by the following groups: *Salmonella enterica* serovar Typhimurium (*Stm*) strain 1344 and flagellin-deficient *Stm* ^Δ*flhD*^ (Dr. Jorge Galan); *Stm* UK-1 wildtype, Δ*wzy*, Δ*waaL*, Δ*waaJ*, Δ*waaI*, Δ*waaG*, Δ*lpxR*, Δ*pagL*, Δ*pagP*, and χ11088 (*Stm*^*ΔlpxRΔpagLΔpagP*^ triple mutant) (Dr. Roy Curtiss III, Dr. Soo-Young Wanda) (38,40); *Pseudomonas aeruginosa* L2 strain (Dr. Barbara Kazmierczak), *Bacillus subtilis* (Dr. Farren Isaacs) and *Listeria monocytogenes* 140203S (Dr. Herve Agaisse).

The following *Stm* strains were constructed on a 1344 isogenic background: *Stm*^*ΔminD*^, *Stm^pBAD::ftsZ^*, *Stm^mreB(K27E)^*, *Stm^mreB(D78V)^*, *Stm^mScarlet-I^*, and *Stm^eGFP^*. In addition, *Stm^ΔWaaG::pBAD-ftsZ^* was generated on the UK-1 background for cryo-ET. See Table S2 for details.

To generate *Stm* MinD deletion and MinD/waaG double deletion mutants in UK-1, the lambda red recombinase system was used. A kanamycin cassette with minD flanking sequences was amplified by primers minDKO-L(GTTTACGATTTTGTAAACGTCATTCAGGGCGATG CGACtgtgtaggctggagctgcttcg) and minDKO-R (GGAGATGTTCTTTAATCGGTTCTTCGCC ATTTTCTcatgggaattagccatggtcca) with pKD4 vector as the template. The PCR product was gel-extracted and electroporated into wild type UK-1 and waaG deletion UK-1 *Stm* competent cells, which were expressing lambda redrecombinase. Kanamycin (Km) resistant strains was plate selected and kanamycin cassette insertion into the minD gene was checked by minD Km insert validation primers, minD-Km-L (ATTTTGTAAACGTCATTCAGGGCG) and minD-Km-R (gcagttcattcagggcaccg). To check the deleted region of minD, primers minD-WT-L (GCTGATCAAAGATAAGCGTACTGA) and minD-WT-R (CGATGCCAGAATACCCAG AATACG) were used.

### Cell culture and transfection

HeLa (CCL-2) and 293T (CRL-3216) cells were purchased from the American Type Culture Collection (ATCC). Cells were grown in DMEM supplemented with 10% (v/v) heat-inactivated fetal bovine serum (FBS) at 37°C in a 5% CO_2_ incubator. Lentiviral (LentiCrisprV2; Addgene Plasmid #52961) or retroviral (pMSCV-puro; Takara 634401) transductions were done by incubating dilutions of 0.45 μm filtered supernatants from transfected 293T cells with 8 μg/mL polybrene for 24 h. For selection of stable transductants, 1 μg/ml puromycin was included. For transient transfections, *Trans*IT^®^-LT1 (MIRUS; MIR 2300) was used according to manufacturer’s instructions. To minimize toxicity in microscopy experiments, 200 ng of DNA was transfected per 24-well cover slip. HeLa cells were stimulated with 500-1,000 U/mL IFN-γ for 18 h.

### CRISPR-Cas9 cell lines & stable complementation

To generate stable gene knockouts in HeLa CCL2 cells, sgRNAs were cloned into pX459 (Addgene Plasmid #62988) per established protocols. 2-4 sgRNAs targeting each gene (200 ng total DNA) were transfected in 24 well plates for 24 h, followed by selection with 1 μg/mL puromycin for 48 h. Surviving cells were expanded into media lacking puromycin for 48 h, then subject to limiting dilution to obtain single colonies. Colonies were screened first by PCR, then by western blot and the genotype of each positive clone determined by Sanger sequencing. The following 10 CRISPR-Cas9 mutants were generated: GBP1^−^/^−^, GBP2^−^/^−^, GBP3^−^/^−^, GBP4^−^/^−^, GBP1^−^/^−^2^−^/^−^ double mutant, GBP^D1q22.2^ septuple mutant (GBP1^−^/^−^GBP2^−^/^−^GBP3^−^/^−^GBP4^−^/^−^GBP5^−^/^−^GBP6^−^/^−^GBP7^−^/^−^), CASP4^−^/^−^, GSDMD^−^/^−^, AOAH^−^/^−^, and RNF213^−^/^−^. A complete list of gRNAs used to generate these CRISPR deletions are provided in Table S3.

In addition, we generated a series of cell lines stably or transiently complemented with GBP mutants, affinity probes or reporters. These included GBP1^−^/^−^ clonal lines complemented with either of the following: EGFP-GBP1, RFP-GBP1, mNG-GBP1, EGFP-GBP1^S52N^, EGFP-GBP1^DD103,108NN^, EGFP-GBP1^D184N^, EGFP-GBP1^C589S^, EGFP-GBP1^α13ARR^, EGFP-GBP1^α13RAR^, EGFP-GBP1^α13RRA^, EGFP-GBP1^α13ARA^, EGFP-GBP1^α13AAR^, EGFP-GBP1^α13AAA^ (R584-586A). Here alanine scanning mutations in the C-terminal polybasic patch (a.a. 584-586) of GBP1 were introduced according to Stratagene Quickchange (Agilent 200523) protocol and confirmed by DNA sequencing. Transfections of plasmids for complementation into GBP1^−^/^−^ HeLa cells were performed using Mirus LTI according to manufacturer protocol. Briefly, 1 μL Mirus LTI was added to 50 μL serum-free DMEM followed by the addition of 500 ng of respective construct. The mixture was allowed to sit for 30 min followed by gentle mixing via pipetting. Cells were selected for hygromycin resistance on the puromycin-resistant GBP1^−^/^−^ HeLa cell background.

We also complemented CASP4^−^/^−^ with EGFP-caspase-4 and GSDMD^−^/^−^ with either EGFP-FL-GSDM EGFP-NT-GSDMD, or EGFP-CT-GSDMD. The latter plasmids were also introduced into CASP4^−^/^−^ and GBP1^−^/^−^ clonal lines as above for fluorescent microscopy.

### Bacterial infections, LDH assay and IL-18 ELISA

For *Stm* infections, overnight bacterial cultures were diluted 1:33 in fresh Luria broth (LB; Thermo 12780029), grown for 3 h before being washed once in PBS and used to infect HeLa cells at 80% confluence with an M.O.I. of 20-50 as indicated. Plates were centrifuged for 10 min at 1000 x *g* and incubated for 30 min at 37°C to allow invasion. Extracellular bacteria were killed by replacing media with fresh DMEM (Thermo 11965092) containing 100 μg/ml gentamicin (Thermo 15710064) for 30 min. Cells were washed 3 times and incubated with 20 μg/ml gentamicin for the duration. To enumerate live bacteria, cells were lysed in PBS + 0.5% Triton X-100 and serial dilutions plated on LB agar. For LDH assay, cell death was measured by CytoTox 96^®^ Non-Radioactive Cytotoxicity Assay (Promega G1780). IL-18 release in supernatants was detected via human IL-18 ELISA kit (Abcam; ab215539) per the manufacturer’s instructions with a detection sensitivity of 8.3 pg/mL.

### CLICK chemistry & metabolic labeling

#### FPP-azide-biotin CLICK chemistry

293E cells (ATCC HEK CRL-1573) expressing GFP or GFP-GBP1 mutant constructs were plated at 2 x 10^5^ cells in each well of a 6-well plate. Cells were then treated with 20 μM Azido farnesyl pyrophosphate (Cayman C10248) for 18 h followed by cell lysis in 500 μL of 20 mM Tris (7.5), 100 mM NaCl, 1% TX-100 buffer containing Roche protease inhibitors. Cells were further sonicated (30% power Virtis Virsonic 600, 3 times for 1 min each) on ice to liberate membrane bound proteins. Lysates were centrifuged at 21,000 *g* for 10 min. Supernatants were transferred to a new Eppendorf tube and were treated with 100 μM Biotin DIBO (Thermo C10412) overnight at room temperature in the dark. 1 μg Roche monoclonal anti-GFP antibody was added to each IP followed by a 2 h incubation at 4°C. 20 μL of pre-equilibrated Protein G Sepharose (Cytiva 17-0756-01) was added to each reaction for an additional 2 h. Beads were pelleted at 4000 *g* for 5 min followed by 8 washes in lysis buffer. Samples were subsequently eluted by 100 μL addition of 2X SDS-Sample Buffer followed by heating at 100°C for 20 min for immunoblotting. Immunoblots were performed with streptavidin-HRP and Roche anti-GFP.

#### KDO-azide Cu^2+^-free CLICK chemistry

The 3-Deoxy- D-manno-octulosonic acid (KDO) of log-phase *Stm* was labelled using CLICK-mediated according to the manufacturer’s instructions (Jena Bioscience). Briefly, an azide modification of the C8-position of KDO with a biorthogonal azido group was introduced to prevent reverse metabolism by KDO-8-P phosphatase. This 8-azido-8-deoxy-KDO modification enabled a biotin group within a dibenzocyclooctynol (DIBO) alkyne dye intermediate (Jena Bioscience CLK-A105P4) to be introduced via Cu(I)-free CLICK chemistry for addition of the Alexa Fluor 594 tag (Jena Bioscience CLK-1295). For infection experiments, we conducted click chemistry reactions on bacteria that had already incorporated fluorescently labelled D-alanine into the underlying peptidoglycan scaffold using a pulse of 500 μM HCC-amino-D-alanine (HADA; MCE HY-131045). Unincorporated DIBO was removed via extensive washing in PBS.

#### Metabolic labeling of Stm peptidoglycan & LPS release in cellulo

Fluorescent blue or red D-alanine analogs (HCC-amino-D-alanine, HADA [MCE HY-131045]; TAMRA 5-amino-D-alanine using 5-Carboxytetramethylrhodamine [ab145438]) was incorporated as described (44). Infection of HeLa cells at MOI 20. After 40 min of infection the media was changed to DMEM with 100 μg/mL gentamycin, after next 1 h to 30 μg/mL gentamycin. Cells were fixed with PFA at 2 h post-infection and immune-stained for GBP1 or LPS. At least 10 GBP1-positive and 10 GBP1-negative fields of view were collected using the DeltaVision^™^ OMX SR Blaze microscopy system (GE Healthcare) in the 3D-SIM mode (512 × 512 pixels, 1 ms exposure, 125 nm step, 8 z slices, 15 images per slice). All images were subjected to processing to widefield image, de-convolution, and maximum intensity projection for semiautomatic analysis in CellProfiler (Broad Institute, Open Scholar, 2021).

### Microscopy

#### 3D-SIM & Multicolor Confocal Microscopy

HeLa cells were grown on 12 mM high performance cover glass #1.5h (Thorlabs CG15KH1) for microscopy of fixed samples. For live imaging, cells were seeded on 4-well chambers (Cellvis C4-1.5H-N) with 1.5 high performance cover glass. Here seeding occurred 48 h prior to imaging to reach 80% confluency on the day of infection. They were treated with IFN-γ for 18-24 h prior to imaging. Bacteria were added to cells as described for infections at an MOI of 20. Images were analyzed on a DeltaVision^™^ OMX SR Blaze microscopy system (GE Healthcare) or a laser scanning confocal model SP8 (Leica).

For ultrafast live imaging, GBP1^−^/^−^ HeLa cells were transfected with RFP-GBP1, induced with 1,000 U/mL IFN-γ for 18 h and infected with EGFP-*S.tm* at MOI 20. After 40 min, the media was changed to DMEM with 30 ug/mL gentamycin and the sample was imaged starting 60 min post-infection at 37°C, 5% CO_2_. Images were collected every 45 s using OMX-SR Blaze microscope (GE) in 3D-SIM 512 x 512-pixel mode at ~180 frames.sec^−1^. Images represent maximum intensity projections of deconvolved z-stacks of Moire fringe patterns (Softworx, GE). Post-acquisition calculations for real-time voxel (boxed) assembly events used Imaris (Oxford instruments) software. When combined with 3D atomic structure volumes of GBP1 (PDB 1F5N) and RFP (PDB 1GGX) calculated via the Voronoi cell algorithm (http://proteinformatics.org/voronoia), the total number of spatially constrained RFP-GBP1 molecules per voxel were counted, along with coat kinetics. Euclidean Voronoi cell algorithms enable the packing densities of all atoms in a deposited protein structure (PDB) to be estimated, accounting for the volume inside an atom’s van der Waals sphere, the solvent excluded volume of the atom, and the position as well as approximate radius of every cavity that can accommodate a water molecule within the protein to yield complete volumetric information (52). In addition, a compensatory ratio of 3D-SIM fluorescence versus EM volumes of GBP1-coated bacteria was included since the former does not resolve objects to the same precision as EM. 3D-SIM volumes were on average 3.02 x larger (*n* = 17) than direct measurements by immuno-EM. Thus, 3D-SIM volumes were divided by 3.0 to correct for fluorescence enhancement. Long-term imaging used low light OMX-SR conventional mode for up to 2.5 hours continuous recording at 5-minute intervals (fig. S3C).

For examining the GBP-COAT_450-708_ complex, we constructed fluorescent fusion proteins GBPs1-4, caspase-4 and GSDMD across different spectral range to accommodate 5 or possibly 6 proteins simultaneously along with *Stm*. A color-coded matrix of 60 proteins was generated by fusing each of the 6 coat proteins to each of the following fluorescent reporters: mAzurite-c1 (Addgene Plasmid #54583), mT-Sapphire-c1 (Addgene Plasmid #54545), pmTurqouise2-c1 (Addgene Plasmid #60560), pmEmerald-c1 (Addgene Plasmid #53975), pmVenus-c1 (Addgene Plasmid #27794), pmOrange-c1 (Addgene Plasmid #54680), pmKeima-Red-c1 (Addgene Plasmid #54546), pmCardinal-c1 (Addgene Plasmid #54799), pmApple-c1 (Addgene Plasmid #54631) and mIFP24-c1 (Addgene Plasmid #54820); E_x_/E_m_ range, 384/450-684/708nm. In brief, GBP1,2,3 and 4 were amplified by PCR with each primer from IFNα-treated HeLa cell cDNA. Caspase-4 and GSDMD were amplified by PCR with each primer from Caspase-4 cDNA (Sino Biologicals #HG11158-M) and pET-SUMO-hGSDMD (Addgene, Plasmid #111559), respectively. Amplicons were cloned into reporter plasmids using *Sal*I/*Bam*HI restriction enzyme digestion. All constructs were sequenced for validation. Multi-arrayed testing found 5 combinations of either pmIFP24-GBP1, pmOrange-GBP2, pmVenus-GBP3, pmEmerald-GBP4, Sapphire-caspase 4 or -GSDMD, or pmOrange-GSDMD could be used successfully with *Stm*.

GBP-COAT450-708 combinations were transfected into the GBP^D1q22.2^ septuple mutant by TranslT-LT1 transfection reagent (Mirus Bio) according to manufacturer’s instructions. After 24 h, cells were activated with IFN-γ for 16-18 h and infected with SPI1-induced *Stm* SL1344 at MOI = 5~10 for 30 min and further incubated with 100 μg of Gentamycin for 50 min to eliminate extracellular bacteria. Five or 6-color fluorescence images were captured on Laser Scanning Confocal Microscope (SP8, Leica) with Z-stacks. A series of lasers were used to excite fluorescence across this broad wavelength and avoid bleed-through as shown in fig. S4E. The corresponding laser parameters (laser, % power, wavelength reception band) used were: pSapphire (Diode 405, 4, 424-444), pmEmerald (WLL 470, 20; 490-520), pmVenus (WLL 516, 6; 535-559), pmOrange (DPSS 561, 25, 566-593), pmIFP24 (HeNe 633, 75, 680-730). Images were analyzed by LAS X software (Leica) for 3D reconstruction.

#### 4Pi single-molecule switching (4Pi-SMS) nanoscopy

For 4Pi-SMS imaging, a customized microscope built using a vertical 4Pi cavity around two opposing high-NA objective lenses as detailed elsewhere (28) was used to capture high-resolution images of the GBP coat complex in IFN-γ-activated HeLa cells. Cell samples were prepared on 25 mm diameter round precision glass cover slips (Bioscience Tools, San Diego, CA) that had been immersed in 1M KOH and sonicated for 15 min in an ultrasonic cleaner (2510 Branson, Richmond, VA). Sequential washes in Milli-Q water (EMD Millipore, Billerica, MA) and sterilization with 70% ethanol was followed drying and poly-lysine coating of coverslips. HeLa cells were grown on coverslips for 24–48 h before fixation in 4% paraformaldehyde (PFA). Anti-GBP1 (1B1; Santa Cruz; 1:500 dilution) and GBP2 (1E5; Origene; 1:200 dilution) antibodies were labeled for 2 h at room temperature by goat anti-mouse Fab AF647 (Jackson ImmunoResearch, PA) and goat anti-rabbit IgG CF660C (Biotium, CA) at 1:200 dilution as described (29).

1B1 antibody was raised against the full-length GBP1 protein while anti-GBP2 targets an N-terminal 17-amino acid peptide epitope. This enabled both antibodies to bind their endogenous GBP targets because the N-terminal LG is oriented towards the top of the coat complex. Specificity was confirmed in IFN-γ-activated GBP^Δ1q22.2^, GBP1^−^/^−^ or GBP2^−^/^−^ HeLa cells as negative controls: all went unlabeled at the single molecule level. At least several hundred GBPs were detectable on cytosolic bacteria using this technique, typically ~400-800 molecules. While this number represents < 2-3% of the total enumerated via cryo-ET in cell-free systems, when 1B1 antibody staining was used on cell-free GBP1-coated bacteria it gave similarly low numbers. Hence, antibody accessibility (due to the densely packed nature of the coat) together with larger secondary antibody detection were limiting factors. Commercially available nanobodies do not currently exist for GBP1 or GBP2. Importantly, however, this comparison discounted bacteria having fewer GBP coat proteins *in cellulo* compared with *in vitro* as both gave similar results when labelled with the same polyclonal antibody.

Sample mounting, image acquisition, and data processing were mostly performed as previously described (29) except that the imaging speed was 200 Hz with 642 nm laser intensity of ~12.5 kW/cm^2^. Typically, 3000×100~200 frames were recorded. DME were used for drift correction. All 4Pi-SMS images were rendered using Point Splatting mode (20 nm particle size) with Vutara SRX 7.0.06 software (Bruker, Germany).

### Purification of recombinant proteins

#### Guanylate binding proteins 1-4 (GBPs1-4) and their mutants, caspase-4^C258A^, and RFP-AtGBPL1

The coding sequences of human GBP1 and its mutants were cloned into a customized vector pCMV-His_10_-Halo-HRV-mRFP-TEV for coating assays. HEK 293F suspension cells (a gift from Dr. James Rothman; mycoplasma-negative) was maintained at a concentration of 0.4x10^6^~4x10^6^ cells/m in Expi293 expression medium (ThermoFisher A1435101). 24 h prior to transfection, cells were seeded at a concentration of 1.2 x10^6^ cells/ml. For transfection, cells were harvested and resuspended in fresh medium at a concentration of 2.5 x10^6^ cells/ml. Cells were transfected by adding pCMV-His_10_-Halo-HRV-mRFP-TEV-containing clones to a final concentration of 1 μg/ml in media containing PEI at a concentration of 5 μg/ml. 24 h after transfection, cells were diluted 1:1 (v/v) with fresh medium containing 4 mM valproic acid and cultured for an additional two days. 2 x 10^9^ cells were harvested via centrifugation (500 x *g*, 10 min), washed once in cold PBS, resuspended in lysis buffer (50 mM HEPES, pH 7.5, 500 mM NaCl, 1 mM MgCl_2_, 10% glycerol, 0.5% CHAPS, 1 mM TCEP) and lysed via sonication. Cells were cleared at 35,000 x *g* for 1 h at 4°C. Supernatant was collected and incubated with 1 ml bed volume of HaloLink resin (Promega G1912) at 4°C overnight with gentle rotation. The resin was sequentially washed twice (10 min each) with wash buffer 1 (50 mM HEPES, pH7.5, 500 mM NaCl, 1 mM MgCl_2_, 10% glycerol, 0.5% CHAPS), wash buffer 2 (50 mM HEPES, pH7.5, 1 M NaCl, 10% glycerol) followed by wash buffer 1.

To elute bound proteins, Halo resin was resuspended in lysis buffer and digested with homemade GST-HRV-His protease overnight at 4°C with gentle rotation. Resin was pelleted and the HRV protease was removed from the supernatant via Ni-NTA beads by affinity chromatography (QIAGEN 30210). Flow through was collected, concentrated, and further purified and buffer-exchanged via size exclusion chromatography (Superdex^®^ 200 Increase; GE Healthcare) equilibrated with storage buffer (20 mM HEPES [pH 7.5], 150 mM NaCl, 1 mM MgCl_2_, 1 mM TCEP). Fractions were analyzed by SDS-PAGE, pooled, concentrated and flash frozen in liquid nitrogen before storing at −80°C. Protein concentration of rRFP-tev-hGBP1 (abbreviated rRFP-hGBP1) was determined via BCA and SDS-PAGE electrophoresis with BSA standards running aside.

Recombinant human GBP1, GBP2, GBP3, and GBP4 as well as the GBP1 mutants (hGBP^1S52N^, hGBP1^D184N^, hGBP1^DD103,108LL^, hGBP1^C589S^ and hGBP1^R3A^) were produced by cloning the respective genes into the mammalian expression vector, pCMV-3Tag1A (Agilent 240195), with an N-terminal FLAG tag. Human caspase-4 ^C258A^ was cloned into pcDNA 3.1/Hygro (Thermo V87020) for N-terminal FLAG attachment. Plasmids were transfected via Mirus LT1 into HEK-293 E cells. Cells were harvested after 14 h and lysed for 2 h in buffer 50 mM Tris, 150 mM NaCl, 5 mM MgCl_2_, 1 mM DTT containing 1% Triton X-100 and protease inhibitor. Supernatant containing the expressed protein passed through anti-Flag M2 affinity gel (Sigma A2220) and bound Flag protein eluted using Flag peptide 150 μg/mL in buffer 50 mM Tris, 150 mM NaCl, 5 mM MgCl_2_ and 1 mM DTT. Further chromatographic purification was undertaken on an AKTA FPLC (GE Amersham) instrument. All recombinant proteins made in human cells were subject to Limulus amebocyte assay (LAL; ThermoFisher A39552) to confirm the absence of LPS contamination (<0.01 EU/mL, lower detection limit).

### Size exclusion chromatography

His_6_-tagged GBP1 or GBP1 mutant proteins were prepared as described above. FPLC was performed on an AKTA system equipped with a Superdex 200 10/300 GL size exclusion column (GE Amersham). After pre-equilibrating the column with 5 volume equivalents of buffer (20 mM Tris (7.5), 140 mM NaCl, 2 mM MgCl_2_), 100 μg of each recombinant GBP1 protein was examined. Column buffer was then exchanged and equilibrated with 20 mM Tris (7.5), 140 mM NaCl, 2 mM MgCl_2_, containing 200 μM GDP-AlF_4_^−^ to test GBP1 assembly in the presence of the transition state analog. Chromatographs were generated by absorbance measured at 280 nm over retention time.

### Thin-layer Chromatography

Thin layer chromatography (TLC) was used to separate GTP, GDP and GMP and performed exactly as previously described (7-9). Briefly, α-[^32^P]GTP (Perkin Elmer BLU006H250UC) hydrolysis by purified recombinant proteins in reaction buffer (20 mM HEPES [pH 7.0], 150 mM NaCl, 5 mM KCl, 1 mM MgCl_2_, 100 μM GTP, 10 μCi α-[^32^P] GTP) was determined at 25°C before quenching the reaction with 142 mM EDTA after 7 h. The resulting products were separated by PEI cellulose (Sigma, Z122882) with fluorescent indicator (UV 254) using 750 mM KH_2_PO_4_ (pH 3.5) as solvent and visualized by autoradiography.

### *Limulus* Amebocyte Assay (LAL) for lipid A detection in OM disruption assays

Overnight-cultured *Stm* was pre-incubated with indicated inhibitors at 37°C with shaking for 2h. After centrifugation by 3,500 rpm room temperature for 20 min, bacteria were washed three times by 10-fold volume of coating buffer (50 mM HEPES (pH 7.5), 150 mM NaCl, 1 mM MgCl_2_ and 1 mM DTT) and finally resuspended by 1 mL of coating buffer containing GTP (2 mM), GTPγS (2 mM), GMPPCP/CppCp (10 mM), GDP.AIF_4_^−^ [2 mM GDP, 200 μM AlCl_4_, 10 mM NaF] or GMP.AIF_3_^−^ (2 mM GMP, 200 μM AlCl_3_, 10 mM NaF]. Bacteria aliquoted were incubated with or without 4 μM of recombinant GBP1 or its mutants at 30°C for 1 h. Supernatants were carefully collected by centrifugation (8,000 rpm, 22°C for 2 min) and diluted 1:1000-1:5000. Released LPS was measured by ToxinSensor^™^ Chromogenic LAL Endotoxin Assay Kit (GenScript L00350) according to manufacturer’s instructions.

### LPS-binding Assays

Human GBP1, GBP2, GBP3, GBP4, GBP1 mutants (GBP1^S52N^, GBP1^D184N^, GBP1^DD103,108LL^, GBP1^R53A^ and GBP1^C589S^) and caspase-4^C258A^ proteins were expressed as Flag-tagged proteins in human embryonic kidney (HEK)-293E cells and isolated from large-scale adherent cell culture using Flag M2 beads as described above. Recombinant human proteins were further purified to single peak via FPLC. Fluorescence anisotropy assays were conducted at 37 °C across different GBP concentrations in binding buffer (50 mM Tris, 150 mM NaCl, 5 mM MgCl_2_, 0.3 mM GDP and AlF_X_ [10 mM NaF, 0.3 mM AlCl_3_], pH 7.0) and Flag- caspase-4^C258A^ (50 mM Tris, 150 mM NaCl, 1 mM DTT, pH 7.0) for 15 min followed by addition of *Salmonella* minnesota LPS-Alexa Fluor 488 to a final concentration 250 nM. After 1 hour incubation with LPS, fluorescent anisotropy values measured by SpectraMax i3x (Molecular Devices).

### Reconstituted Coat Assays

Wild-type *Stm* and *Stm* mutants were freshly streaked on LB plates. A single fresh colony of bacteria was cultured in LB medium overnight at 37°C. Before the coating assay, bacteria were diluted 1/60 in fresh LB medium, cultured for additional 2 h and harvested via centrifugation (4000 *g*, 5 min at 22°C). To remove shed LPS which inhibits rRFP-hGBP1 targeting, bacteria was washed twice in coating buffer (50 mM HEPES [pH 7.5], 150 mM NaCl, 1 mM MgCl_2_) and further diluted to an optical density (OD _600_) of 0.1 in coating buffer. Immediately before coating, rRFP-hGBP1 was thawed at room temperature and centrifuged at 12,000 *g* for 15 min to remove any insoluble aggregates. For coating, 2 μM rRFP-hGBP1 and 2 mM GTP were added to each 0.1 OD of bacteria, gently mixed and incubated for 1 h at 22°C before imaging.

To image rRFP-hGBP1-coated bacteria, coating reactions (20 μL) were transferred to 384-well glass bottom plate (Cellvis; P384-1.5H-N). The plate was centrifuged at 2000 *g* for 1 min to collect all liquid to the bottom of the well. All images were acquired using a Leica SP8 laser-scanning confocal microscope with a 63×/1.40 oil immersion objective. Focal plane was set to the bottom of the well. RFP was excited at 561 nm and detected at 590–610 nm. The optical slices were acquired in confocal mode (1 Airy unit) with an average of six scans. Images were collected in a 512 × 512 format. Image analysis was performed with FIJI/ImageJ.

For coat complex assembly, samples were doubly diluted from 2 μM rRFP-GBP1 until loss of coating to establish a coating curve. The observed behavior was plotted with best-fit interpolation and a sigmoidal curve emerged. Curve fitting together with regression analysis found half maximal and Hill slope values using Graph Pad Prism 9.1.1.

### Reconstituted Killing Assays

For bacterial killing assays, *Stm* were grown to mid-log phase in LB medium, washed, and incubated with 5 μM hGBP1 in coating buffer (50 mM HEPES pH 7.4, 150 mM NaCl, 5 mM MgCl_2_) with or without 2 mM GTP, 2 mM GTPγS or GDP.AIF_4_^−^ ([2 mM GDP, 200 μM AlCl_3_, 10 mM NaF]). *Stm* was then pelleted and re-suspended in Buffer A ([50 mM MES pH 6.0, 100 mM potassium gluconate (KGluc)]) containing 5 μM rAPOL3 and incubated at 37°C for 1 h prior to plating on LB agar to enumerate colony forming units (CFU) in triplicate.

### In vitro Phase Separation

Protein aliquots of rRFP-GBP1 and rRFP-GBPL1 (7) were thawed at room temperature, centrifuged at 14,000 × *g* for 5 min to remove any aggregated protein. Droplet formation was induced by diluting protein to low salt buffers (50 mM HEPES, pH7.5, 150 mM NaCl, 1 mM MgCl_2_) by mixing with various volumes of no salt buffer (20 mM HEPES [pH 7.5], 1 mM TCEP) and analyzed in chambered coverglass (Grace Bio-labs) using a Lecia SP8 laser scanning confocal at 63×/1.40 magnification. Addition of Ficoll at 5%, 10%, 15%, 20% w/v had no effect on rRFP-GBP1.

### Electron Microscopy

#### Negative-stain electron microscopy

To visualize rRFP-GBP1 on minicells, 100 μL minicells were washed and dissolved in the HEPES buffer (pH 7.4) to the OD_600_ equal to 0.1. The minicell suspensions were incubated with rRFP-GBP1 with a final concentration of 2 μM in the presence/absence of GTP at 22°C for 1 h. The mixture was concentrated to 10μL for further TEM observation. 5 μL RFP-GBP1 coated and non-coated minicells were loaded onto glow-discharged carbon film coated cooper EM grids (EMS, CF400-Cu-50) for standing 1 min. The EM grid was washed and stained with 2% uranyl formate for 30 sec. The grid was blotted by filter paper (Whatman FILTER PAPERS 2; 1002-090) and dried for 1 min. Grids were examined in JEOL1400 plus electron microscope with acceleration voltage of 80 kV.

### Cryo-electron Tomography

#### Cell Seeding on EM Grids

Pre-treatment of EM grids. EM grids (Quantifoil R1/4 gold 200 mesh, Q250AR-14) were placed onto 18 mm^2^ cover-glass that had been washed and stored in 100% ethanol. They were then placed into glass bottom dishes (Cellvis, 35 mm dish with 20 mm micro-well cover glass) and treated with 70% ethanol under UV illumination for 10 min at 22°C. EM grids were washed 6 times with sterile and degassed water before treatment with 0.05 mg/mL collagen I (Gibco, A10644-01) for 60 min in a 37°C incubator followed by washing in degassed/distilled water. Collagen-coated grids were incubated in DMEM medium overnight at 37°C incubator with 5% CO_2_ for the further use.

The GBP^1q22.2^ septuple mutant was cultured in 10 cm dish to the density of 4 x 10^6^ cells per dish. HeLa cells were transfected with EGFP-GBP1 plasmid using Mirus LT1 kit for overnight expression. Cells were subsequently washed by PBS and treated with 0.05% Trypsin-EDTA for detachment. Detached HeLa cells were centrifugated and resuspended with DPBS buffer before filtering via 40 μm nylon mesh filter (Fisher Scientific, 22363547) to remove larger debris. Filtered HeLa cell suspensions were transferred into 5 mL polystyrene round-bottom tube (Corning, 352235) with cell-strainer cap for FACS sorting. GFP-positive cells were sorted into collection tubes, centrifugated and resuspend to a final cell density of 2 x 10^4^ cells/mL in DMEM supplemented with 10% fetal bovine serum plus 100 μg/mL Pen Strep (Gibco, 15140-122). EGFP-GBP1-expressing cells were seeded onto pre-treated EM grids and further incubated for overnight. For the infection assay, *Stm*^*pBAD-ftsZ*^ induced by by 0.2% L-arabinose were cultured to a density of OD_600_ 1.0 and infected at MOI 200. EM grids coated with GBP^Δ1q22.2^ HeLa cells and bacterial cells were further centrifuged at 800 *g* for 5 min for spinning bacteria onto the HeLa cell surface. EM grids were transferred into 37°C incubator for additional 20 minutes before washing 3 times with DPBS and incubated with complete DMEM plus 100 μg/mL gentamycin for an additional 1 h infection.

For preparation of minicells from *Stm*^ΔminD^, bacterial cultures were grown overnight at 37°C in LB medium in the presence of 100 μg/mL ampicillin. 10 mL bacterial overnight cultures were added into 1 L fresh LB medium in the presence of ampicillin and were grown to late log phase. Batch cultures then underwent 3-step centrifugation at: (i) 6,240 *g* (Beckman coulter, Avanti JXN-26, rotor, JLA-8.1000) for 10 min to remove parental rod-shaped bacilli; (ii) 24,820 *g* (rotor, JLA-16.250) for 10 min to collect minicell fractions which were resuspended in HEPES buffer (pH 7.4) and filtered by 0.45 μm PVDF membrane (Merck Millipore, SLJVM33RS); (iii) 20,000 *g* (rotor, JA-25.50) for further 10 min. Minicell fractions were adjusted to OD_600_ 1.0 for rRFP-GBP1 *in vitro* coating.

For preparation of minicells from *Stm*^*ΔwaaG::pBAD-ftsZ*^, fresh cultures were grown in LB medium containing 100 μg/mL ampicillin and 0.2% L-arabinose for a continuous *FtsZ* induction at 37°C for 20 h. *Stm*^*ΔwaaG::pBAD-ftsZ*^ minicells were collected as described above.

#### Vitrification and Cryo-correlative Fluorescence Light Microscopy (CLEM).

EM grids seeded with EGFP-GBP1-expressing HeLa CCL2 cells were pre-screened using fluorescent light microscopy, clipped on a custom manual plunger, blotted using Whatman #1 filter paper (GE, 1001-110), and frozen in liquid ethane by the plunger. Frozen grids were transferred onto a cryo-stage and clipped within the O-ring and C-ring (ThermoFisher Scientific, cryo-FIB autogrid and C-clip, respectively). Cryo-CLEM (Leica Microsystems EM Cryo-CLEM microscope) was performed as previously described (7). Grids were transferred into Leica-CLEM cartridge docked at a pre-cooled shuttle docking station and then transferred onto a cryo-stage equipped with a pre-cooled 40× objective lens. A spiracle 4 × 4 grid region was selected, and 16 sub-regions were imaged. Montage EGFP-positive images were acquired on the selected area using the Z-stack mode within a stepwise of 0.35 μm. This montage served as the navigating map in subsequent milling assays by the focused ion beam method.

#### Milling Lamella by Focused Ion Beam (FIB)

Cryo-CLEM demarcated grids were transferred into cryo-DualBeam microscope equipped with cryo-stage and cryo-transfer shuttle systems (Thermo Fisher Scientific, Aquilos cryo-FIB focused ion beam/scanning electron microscope). The lamella was prepared according to Aquilos cryo-FIB protocols. The sample on the O-ring side of the grid was sputter-coated with platinum (1 kV, 30 mA, 10 pa, 15 s) to increase sample conductivity during FIB milling. MAPS software was used to capture an EM grid montage in the electron beam mode before importing the fluorescent montage from cryo-CLEM to generate a merged map to establish potential targets for milling lamella. Eucentric height of the targeting area was refined and stage tilting angle determined at 16° for lamella. At target positions on the grid organometallic platinum coating was sprayed for 5 seconds by a gas injection system (ThermoFisher Scientific, GIS).

Initially, rough milling was started from the topside with 300 pA current and continued in a small stepwise manner. Concurrently, SEM images were taken to observe intracellular elongated bacteria. Rough milling stopped on the topside when elongated bacteria were closely visible. Rough milling on the bottom-side began with 300 pA current in a large stepwise manner. When the lamella thickness arrived to 1.5 μm distance, the milling current was changed to 100 pA until the lamella thickness was ~0.8 μm. Thereafter fine milling focused primarily on the bottomside at < 50 pA. An electron beam was used to monitor the milling process at 5 keV and 13 pA. A final fine milling was done on both sides of the lamella. Subsequently the grid was sputter-coated with platinum (15 mA, 10 Pa, 10 s) to increase conductivity of milled lamella. The grid was transferred from the FIB instrument using the transferring shuttle system and stored (SubAngstrom, Pin type grid box) in liquid nitrogen for further data collection or immediately re-confirmed by CLEM for correct positioning of GBP1-coated bacteria.

#### Cryo-electron Tomography and Image Processing

Tomography data for the FIB-milled lamella sample were collected using a Titan Krios G2 transmission electron microscope (Thermo Fisher Scientific) equipped with a 300 kV field-emission gun, a Volta phase plate (phase shift located around 1/2 pi), a Quantum post column energy filter (20-eV slit) and a Gatan K2/K3 summit direct detection camera. We first navigated the stage to the lamella and collected a montage at medium magnification (2250 x); we used ImageJ to overlap the TEM montage with the map taken by a 2nd CLEM. Three ice deposits as well as a double-membrane vesicle on the lamella were used as shared markers to overlay these two maps. One of the successful targets with a GFP signal was located beneath the largest ice deposit and close to the lamella edge. The stage was moved to the right of the target with fluorescent signal to collect tilt series. The images were taken in a dose-fractionated mode at near focus using SerialEM software. The resulting physical resolution is 0.45 nm/pixel. A total dose of 80 e/Å^2^ distributed among 33 tilt images was undertaken covering angles from 39° to −57° at tilting step of 3° and starting the first tilt series at −9° in a continuous data collection mode. The dataset for the minicell were taken a dose symmetric scheme without using phase plate. The tilt series for minicells sample were taken at the physical resolution of 0.14 nm/pixel starting from 0° and covered angle ranges of 51° to -51° with 3° increments, resulting in a total dose of 100 e/Å^2^.

Collected dose-fractioned data were subjected to motion correction for generating drift-corrected image stack files. Stack files were aligned using patch-tracking function of IMOD. 3D tomograms were reconstructed from aligned stack files by SIRT (Simultaneous Iterative Reconstruction Technique) method using Tomo3D. For reconstruction of binned tomograms, the aligned tilt series was scaled to 3.6 nm as pixel size. IMOD was used for visualizing the tomogram. Surface rendering of tomogram was done with EMAN2.23, and refined with UCSF chimera. Briefly, for ribosomes, a template-matching strategy was performed to determine all ribosome coordinates and orientations. A mammalian 80S ribosome structure (EMD-3418) determined by Volta phase plate cryo-ET, and bacterial ribosome cryo-EM structure (EMD-0076) (53) were low-pass filtered to 20 angstrom and scaled to 3.6 nm/voxel to match to the tomograms. EMAN2.3 was employed to do segmentation on membrane, vesicles and GBP1 coat features. The cryoEM map of type 3 secretion system (T3SS) needle complex from *Salmonella typhimurium* (EMD-11781) (54) was scaled to 3.6 nm/voxel and fitted into the position of T3SS in the tomograms.

#### Sub-tomogram averaging

TomoSegMemTV and PySeg packages were used for sub-tomogram analysis. For initial membrane segmentation, 11 tomograms from *Stm*^*ΔminD*^ dataset and 27 tomograms from *Stm*^*ΔwaaG::pBAD-ftsZ*^ with a binning factor of 8 were selected for membrane segmentation by using TomoSegMemTV (55). PySeg package was used for tracing and picking the particles located on the membrane based on Discrete Morse theory (56). The initial orientation of the particles perpendicular to the membrane was determined by PySeg. The coordinates of picked particles were multiplied by 4 to obtain the particles coordinates information in the CTF corrected and weight back projection reconstructed tomograms within a binning factor of 2. Sub-tomograms were extracted and were initially aligned based on the feature of outer membrane (OM) by Relion package v3 (57).

After the OM was well aligned, general classification function in the PySeg was performed to distinguish membrane and non-membrane features. Classes showing membrane features were merged to have a further general classification by applying a cylinder mask on the top of the membrane. The classification results showing the extra density of GBP1 on top of the membrane resulted in 21,587 particles obtained for further constrained refinement and classification by the Relion package. During the next step of Relion classification, 4 classes resulting in 12,858 particles were averaged by the constrained refinement with molecular masks that was used to remove overlapping density of GBP1 (Fig. S8). During the post-processing, the final density map was generated with Relion using a B-factor of −1000 and a low-pass filter of 10 Å. Resolution estimation and Fourier shell correlation (FSC) for both dimeric and monomeric masks along with their controls are shown in Fig. S9.

For distance detection of the full coat complex of mRFP-GBP1, OM and GBP1 density were manually segmented via IMOD drawing contours. Mtk function was used to detect the shortest distance between the density of the GBP1 GTPase domain and the density of the LPS outer leaflet. 11,889 GBP1 protomers on the ΔminD minicell having wild type LPS; 12,487 GBP1 protomers on the ΔwaaG minicell having O-antigen and outer-core truncated LPS; 6,107 GBP1 protomers on the ΔwaaG outer membrane vesicle (OMV) were detected and analyzed by the Graphpad Prism9 package. For distance detection of the washed coat complex of mRFP-GBP1 on both minicells and OMVs, GBP1 density in the 3D tomogram was drawn using IMOD manually. Neural-network-based segmentation on both washed minicell and washed OMV was performed by EMAN2.23 and further refined by UCSF Chimera.

### Computational modeling of human GBP1

We enlisted AlphaFold2 to predict functional homologs of the human GBP protein family. Notably, the GBP5 protein (AF-A0A2K5PG15) exhibited an elongated conformer resembling some of the structural features revealed in the sub-tomogram averaging of human GBP1. Recent findings suggested that GBP5 lacking its C-terminus can form dimers in the presence of GDP.AIF_3_^−^ (PDB:7E5A).

Based on this dimeric form of truncated GBP5, we constructed a pseudo-model representing dimeric GBP1 in its activated state. First we aligned the N-terminal domain and MD region from the human GBP1 monomer X-ray crystal structure (6K1Z) using the MatchMaker function in the Chimera package; it revealed a 147-degree rotation and a -14.7 angstrom shift along the axis of the GTPase domain when compared to one of the monomer subunits of the GBP5 dimer (PDB:7E5A). Then we aligned the C-terminal region of GBP1 with the predicted GBP5 monomer (AF-A0A2K5PG15) using Chimera. Lastly, pseudo-models of an open GBP1 conformer were built via AlphaFold2 and docked into our low-resolution sub-tomogram averaged GBP1 structures using Chimera. It yielded elongated GBP1 monomer and dimer models corresponding to native structures obtained from our monomer and dimer masks.

### Sequential wash method for reducing GBP1 crowdedness

After reconstituting the coat complex, we collected untagged GBP1 coated minicells via centrifugation at 14,000 *g* for 5 mins (Eppendorf Centrifuge 5420, SN, 5420JQ303731) at room temperature. The minicell pellets were washed in the coating buffer (50 mM HEPES [pH7.5], 150 mM NaCl, 1 mM MgCl_2_) by gently pipetting the solution 10 times. The GBP1-coated minicells were then collected again via centrifugation as above and subjected to the same washing treatment. This was repeated one more time for a total of 30 pipette-aided washes and removal of the freed GBP1. The coated minicells were then dissolved in the coating buffer and ready for cryo-ET sample preparation. This method proved highly efficient, reducing crowdedness from ~12,000 GBP1 molecules per minicell to an estimated ~100-120 molecules per minicell (>100-fold depletion) based on direct counts of segmented tomograph series.

### Ni-NTA-Nanogold Labeling

A his-tag containing 6 histidine residues (His_6_) with a GSG linker was fused to the N-terminus of GBP1. Purified His_6_-GBP1 diluted to 2μM in the coating buffer with 1mM GTP was a mixture with bacterial minicell suspension as the reconstitution assay described. After the full coatomers formed, the cell suspension was centrifuged at 6,200 g and 4 degrees for 5 minutes. The pellets containing bacterial minicell and GBP1 coat were dissolved in the blocking-binding buffer ( 20 mM Tris-HCl, 150 mM NaCl, 0.1% Tween 20, 5% BSA, 1mM MgCl2) for 10 minutes incubation at room temperature. 1.8 nm Ni-NTA-Nanogold beads (Nanoprobes) diluted 1:10 were added in the blocking-binding buffer and incubated for 10 minutes. The suspension underwent centrifugation at 6,200 g for 5 minutes and was washed 3 times in 50 mM Tris-HCl, 100 mM Imidazole, 1 mM MgCl2, 0.1% Tween 20 and 300 mM NaCl (pH 7.4). The minicell pellets were finally dissolved into the coating buffer and transferred onto glow-discharged carbon-coated copper grids for cryo-ET sample preparation.

### Disulfide cross-linking assay

For testing the open conformer model of GBP1, we undertook a Cysteine replacement strategy for cross-linking. Six residues (Glu-389, Ile-369, Glu-366, Ile-365, Arg-362, and Glu-361) present in the GBP1 α7 helix of the middle domain and face towards the α12 helix in GED domain were chosen as prospective Cys-Cys pairs. We measured the distance between the Cβ atoms of the above-selected residues and their neighboring residues (Lys-520, Ser-523, Glu-526, His-527, Gln-530, and Glu-533) located in the α12 in the GED domain and picked the closest neighbor residues for cross-linking candidates (between 2 to 7 Angstroms apart). After mutating these residues to Cysteines, we directly visualized their targeting to cytosolic *Stm in cellulo* (Fig. S13A,B). This confirmed no gross structural alterations that would interfere with targeting. We chose the middle R362C-E526C mutant with the shortest Cys-Cys interbond length (2 Å) to make recombinant RFP-GBP1 for direct cell-free coating assays. It was examined by *in vitro* reconstitution assays in the presence or absence of DTT, a reducing agent. Specifically, Cys-paired GBP1^R362C-E526C^ tagged with mRFP were purified and mixed with bacteria (OD = 0.1) in coating buffer (HEPES buffer with 150 mM NaCl, 1 mM MgCl_2_, pH 7.4). Reconstitution assays under reducing conditions was performed by adding a final concentration of 1 mM DTT into the coating buffer.

### In silico protein sequence analysis

For lipid-binding motifs in human GBP1 compared with other related GTPases, evolutionary tree history was inferred using the Maximum Likelihood method and JTT matrix-based model. A bootstrap consensus tree inferred from 10,000 replicates was taken to represent the evolutionary history of the taxa analyzed. Branches corresponding to partitions reproduced in < 50% bootstrap replicates were collapsed. Initial tree(s) for the heuristic search were obtained automatically by applying Neighbor-Join and BioNJ algorithms to a matrix of pairwise distances estimated using the JTT model, and then selecting the topology with superior log likelihood value. A discrete Gamma distribution was used to model evolutionary rate differences among sites (5 categories (+G, parameter = 2.8088)). Evolutionary analyses were conducted in MEGA X.

### Statistics

Data were analyzed by GraphPad Prism 9.1.1. and Excel v16.54 software. Unless otherwise indicated, statistical significance was determined by t-test (two-tailed) or one-way ANOVA (Dunnett’s multiple comparison tests) with Bonferroni *post-hoc* test.

## Supplementary Material

abm9903.v2 - Movie S1

abm9903.v2 - Movie S2

abm9903.v2 - Movie S3

abm9903.v2 - Movie S4

abm9903.v2 - Movie S5

abm9903.v2 - Movie S6

7

## Data Availability

Sub-tomogram averages of the native GBP1 dimer and monomeric subunit of the dimer have been deposited in the wwPDB Deposition and Annotation deposition system (EMD-43091, EMD-43153). Raw EM images from tilt series and segmentation have been deposited in EMPIAR (EMPIAR-11822). AlphaFold2 rendering of extended GBP1 conformers have been deposited in Zenodo (accession code 10429400). All other data are available in the main text or the supplementary materials.
