# Supplementary material for: Native architecture of a human GBP1 defense complex for cell-autonomous immunity to infection
Source: Science. Author manuscript; Available in PMC 2025 May 20. (PMC12091997; doi:10.1126/science.abm9903)
Supplement: 7 [file NIHMS2075077-supplement-7.pdf]

Science
AAAS

Supplementary Materials for

Native structure of a human GBP1 defense complex for cell-autonomous immunity to infection

Shiwei Zhu, Clinton J. Bradfield, Agnieszka Mamińska, Eui-Soon Park, Bae-Hoon Kim, Pradeep Kumar, Shuai Huang, Minjeong Kim, Yongdeng Zhang, Joerg Bewersdorf , and John D. MacMicking.

Corresponding author: john.macmicking@yale.edu

The PDF file includes:

Figs. S1 to S13
Tables S1-S4
Captions for Movies S1-S6

Other Supplementary Materials for this manuscript include the following:

MDAR Reproducibility Checklist
Movies S1-S6

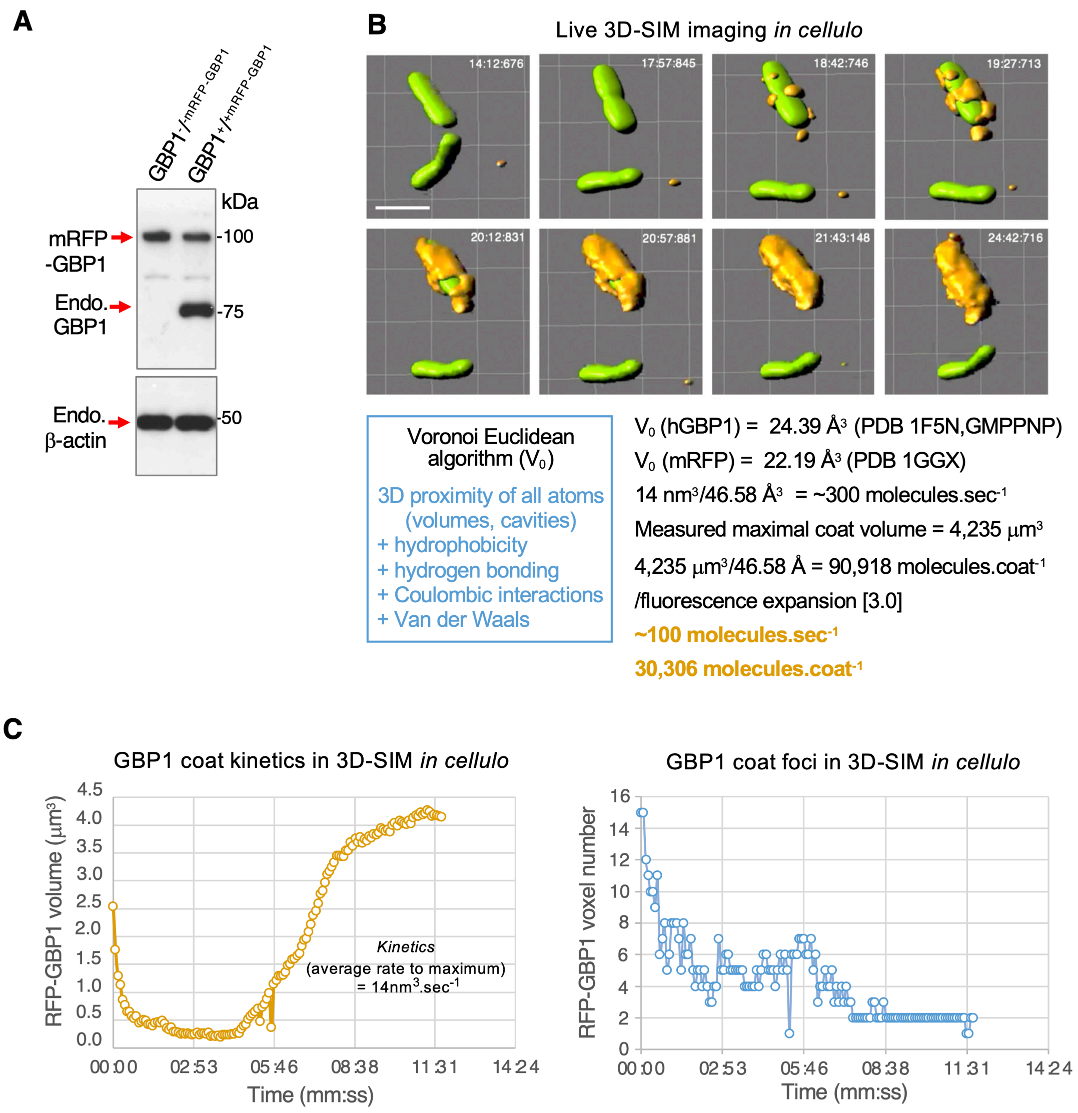

Fig. S1. Assembly characteristics of the hGBP1 coat complex inside human cells.
(A) Stable reconstitution of the mRFP-GBP1 tagged protein in wild-type and GBP1$^{-/-}$ HeLa cells. 1,000 U/ml IFN-γ added for 18 h before detecting endogenous GBP1 and the tagged protein via anti-GBP1 antibody. **(B)** Live 3D-SIM imaging of EGFP-expressing *Stm* 1344 being coated by RFP-GBP1 at 2 h post-infection. 1 of 6 similar 3D-SIM live imaging videos. Volumetric and velocity measurements calculated by Imaris software (below) that incorporate Voronoi protein algorithms and 3-fold fluorescence enhancement, the latter derived from direct EM versus fluorescence measurements of bacteria (n = 17). Scale bar, 2 µm. **(C)** The number of individual voxels diminished as the coat coalesced into a single polymeric platform on the bacilli shown in **(B)**.

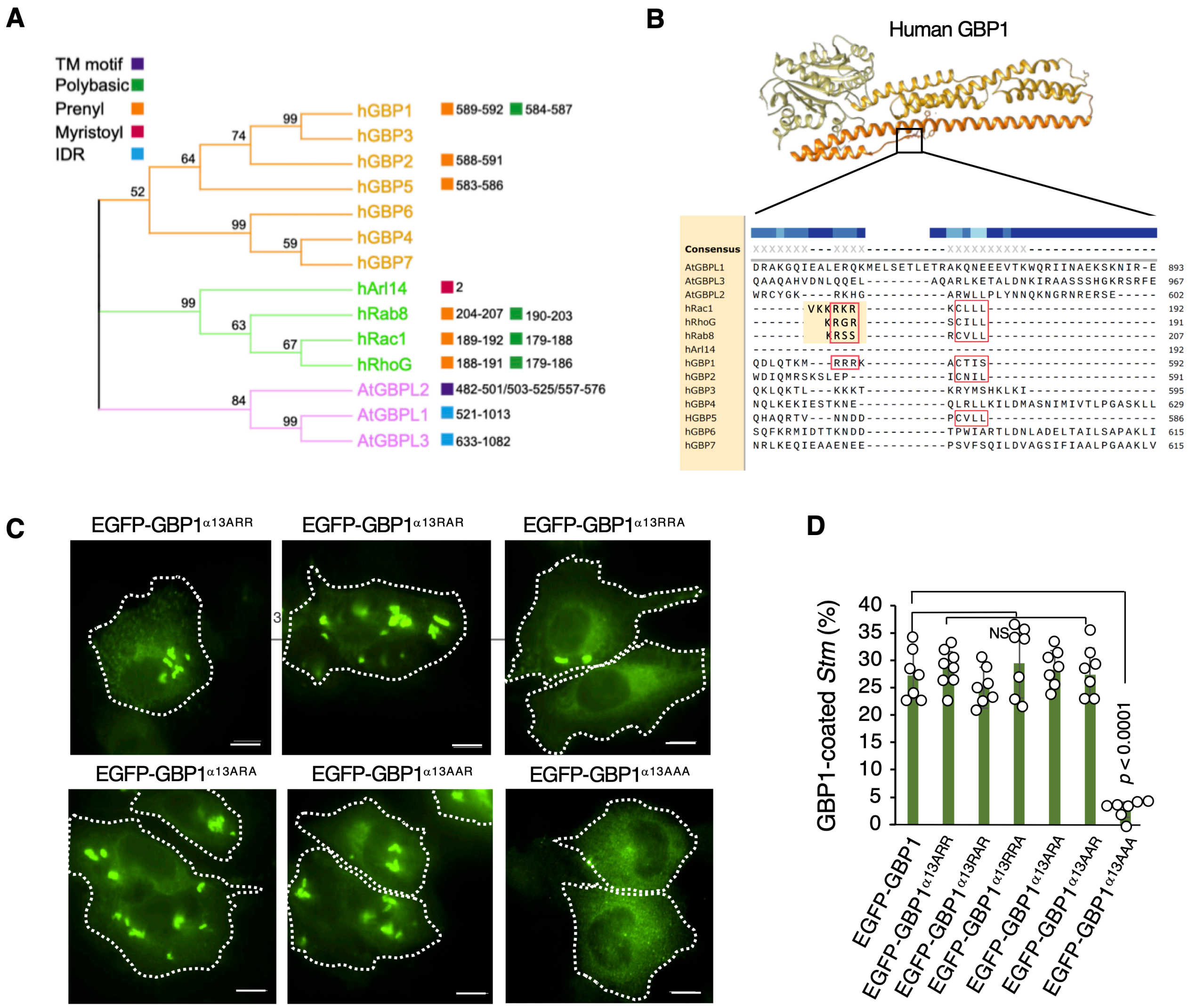

Fig. S2. Determinants of C-terminal human GBP1 anchorage to the bacterial OM.

(A) Evolutionary tree of lipid-binding motifs in human GBP1 versus other related GTPases inferred via Maximum Likelihood methods and a JTT matrix-based model. Percentage of replicate trees where taxa clustered in 10,000 bootstrap replicates depicted next to branches. **(B)** Position of RRR and CaaX motif within GBP1 versus other GTPases in the phylogenetic tree. Yellow box for H-Ras GTPases transposed to show the presence of their C-terminal polybasic patch aligned with GBP1. **(C)** Alanine scanning mutagenesis of the C-terminal polybasic patch (a.a. 584-586; RRR) of EGFP-fused GBP1 mutants introduced into HeLa cells. **(D)** Quantitation from 7 InCell-imaged regions covering >100 bacteria/group. EGFP-GBP1 wild-type cells included as a positive control. Significance determined by one-way ANOVA with Bonferroni *post-hoc* test. 1 of 4 similar experiments shown.

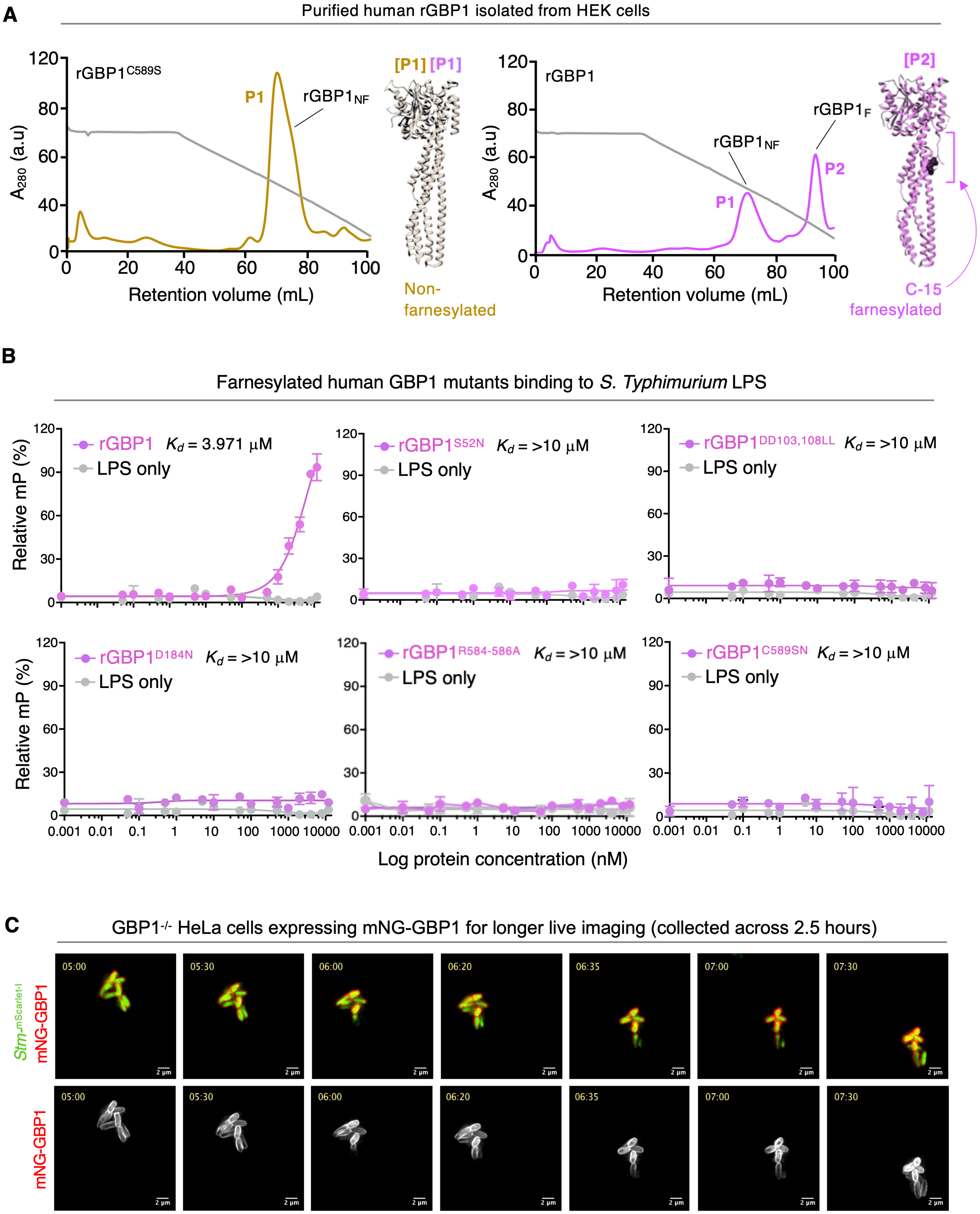

A
Purified human rGBP1 isolated from HEK cells
rGBP1C589S
P1
rGBP1NF
[P1] [P1]
Non-farnesylated
A280 (a.u)
Retention volume (mL)
rGBP1
rGBP1F
rGBP1NF
P1
P2
[P2]
C-15 farnesylated
B
Farnesylated human GBP1 mutants binding to S. Typhimurium LPS
rGBP1 Kd = 3.971 µM
LPS only
rGBP1S52N Kd = >10 µM
rGBP1DD103,108LL Kd = >10 µM
rGBP1D184N Kd = >10 µM
rGBP1R584-586A Kd = >10 µM
rGBP1C589SN Kd = >10 µM
Relative mP (%)
Log protein concentration (nM)
C
GBP1-/- HeLa cells expressing mNG-GBP1 for longer live imaging (collected across 2.5 hours)
StmmScarlet-I
mNG-GBP1
05:00
05:30
06:00
06:20
06:35
07:00
07:30

Fig. S3. LPS binding of farnesylated recombinant GBP1 purified from human cells and live imaging of GBP1 coating over longer timescales.

(A) Isolation of lipidated human RFP-GBP1 for coat complex studies from human HEK cells. Superdex-600 FPLC preparative profiles of isolated peaks containing farnesylated (peak 2; P2) and non-farnesylated (peak 1; P1) species; rRFP-GBP1^{C589S} served as an internal non-farnesylated control. Right, position of the C-15 farnesyl moiety at the C-terminal branch in the 1F5N/6K1Z crystal structure. **(B)** *Salmonella* minnesota LPS-Alexa Fluor 488 (ThermoFisher) binding curves for farnesylated recombinant human GBP1 mutants in fluorescence anisotropy assays. Y-axis, % of maximal polarization (mP). Mean ± SD determined in triplicate for each protein concentration. Representative of 3 independent experiments. **(C)** Live widefield imaging of GBP1$^{-/-}$ HeLa CCL2 cells transfected with mNeonGreen-GBP1, induced with 1000 U/ml IFN-γ for 18 h and infected with mScarlet-i-*S.tm* at MOI 20. After 40 min of infection the media was changed to DMEM with 30 μg/ml gentamycin and the sample was imaged starting ~5 h post infection. Images were collected every 5 min and presented as maximum intensity projections of deconvolved z-stacks (Softworx, GE). mScarlet was chosen to avoid photobleaching over a long period and has been pseudocolored in green (with mNeonGreen-GBP1 switched to red) to depict rod-shaped bacilli more easily *in cellulo*.

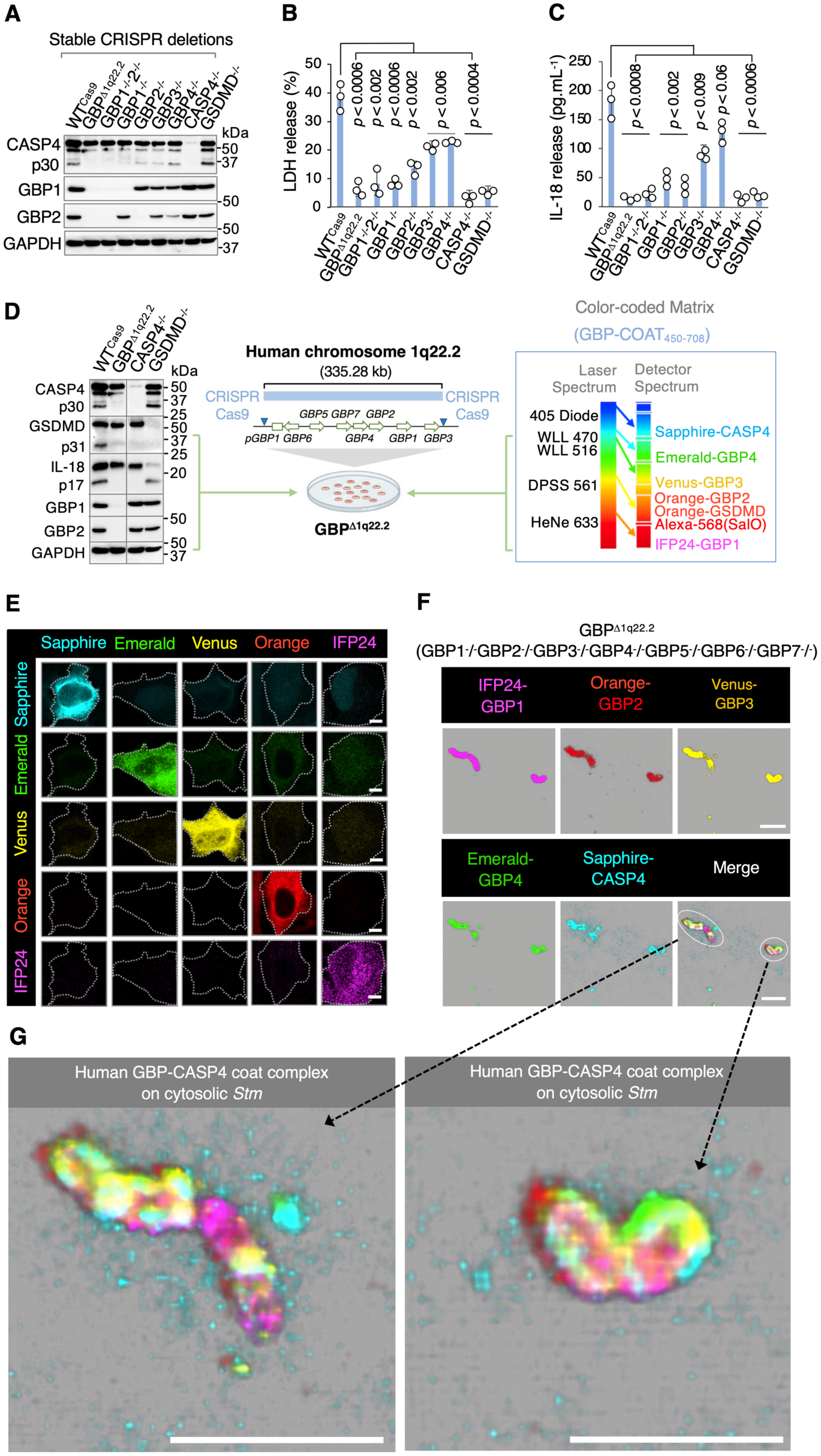
A
Stable CRISPR deletions
CASP4
p30
GBP1
GBP2
GAPDH
kDa
B
LDH release (%)
C
IL-18 release (pg.mL-1)
D
Human chromosome 1q22.2
(335.28 kb)
CRISPR Cas9
GBPΔ1q22.2
Color-coded Matrix
(GBP-COAT450-708)
Laser Spectrum
Detector Spectrum
405 Diode
WLL 470
WLL 516
DPSS 561
HeNe 633
Sapphire-CASP4
Emerald-GBP4
Venus-GBP3
Orange-GBP2
Orange-GSDMD
Alexa-568(SalO)
IFP24-GBP1
E
Sapphire Emerald Venus Orange IFP24
F
GBPΔ1q22.2
(GBP1-/-GBP2-/-GBP3-/-GBP4-/-GBP5-/-GBP6-/-GBP7-/-)
IFP24-GBP1
Orange-GBP2
Venus-GBP3
Emerald-GBP4
Sapphire-CASP4
Merge
G
Human GBP-CASP4 coat complex on cytosolic Stm
Human GBP-CASP4 coat complex on cytosolic Stm

Fig. S4. Functional coat partners and 6-member COAT$_{450\text{-}708}$ reconstitution system. (A) (Top) Immunoblot of cleaved (p30) subunit of human caspase-4 in stably generated CRISPR/Cas9 HeLa cell lines activated with 500U/mL IFN-γ for 18 h before *Stm* infection. Endogenous GBP1, GBP2 and GAPDH shown as controls. 1 of 3 similar experiments shown. **(B)** LDH assay for pyroptotic cell death in stably generated CRISPR/Cas9 HeLa cell lines treated as in A. **(C)** IL-18 release detected by ELISA in stably generated CRISPR/Cas9 HeLa cell lines treated as in A. Representative of 4-6 independent experiments performed in triplicate. Significance determined by one-way ANOVA with Bonferroni *post-hoc* test. **(D)** (Left) Immunoblot of cleaved GSDMD p31 N-terminal fragment and cleaved p17 IL-18 fragment for phenotypic comparison of GBP$^{\Delta 1q22.2}$ versus CASP4$^{-/-}$ and GSDMD$^{-/-}$ cells. Caspase-4 p30, GBP1, GBP2 and GAPDH included as well from (A) above for the same samples. (Center, Right) Introduction of the GBP-COAT$_{450\text{-}708}$ plasmid array into these GBP$^{\Delta 1q22.2}$ cells depicting the removal of the complete human *GBP* gene cluster arrayed on chromosome 1q22.2 to reconstitute the coat complex. **(E)** Establishing laser power and imaging conditions for multicolor confocal microscopy to reduce bleed-through from adjacent channels for 5-color imaging. HeLa cells simultaneously transfected with all 5 fluorescent protein plasmids used for tagging coat proteins and imaged across the 424-730 wavelength reception spectrum. Scale bar, 2 μm. **(F)** Multi-color confocal imaging of the coat complex assembling on the same cytosolic bacilli in IFN-γ-activated GBP$^{\Delta 1q22.2}$ HeLa cells. Individual channels with convergent assembly denoted by dashed circles. Scale bar, 2 μm. **(G)** Enlargement of the reconstituted coat complex in dashed circles depicting sub-regions occupied by different coat proteins on the bacteria surface. Scale bar, 2 μm.

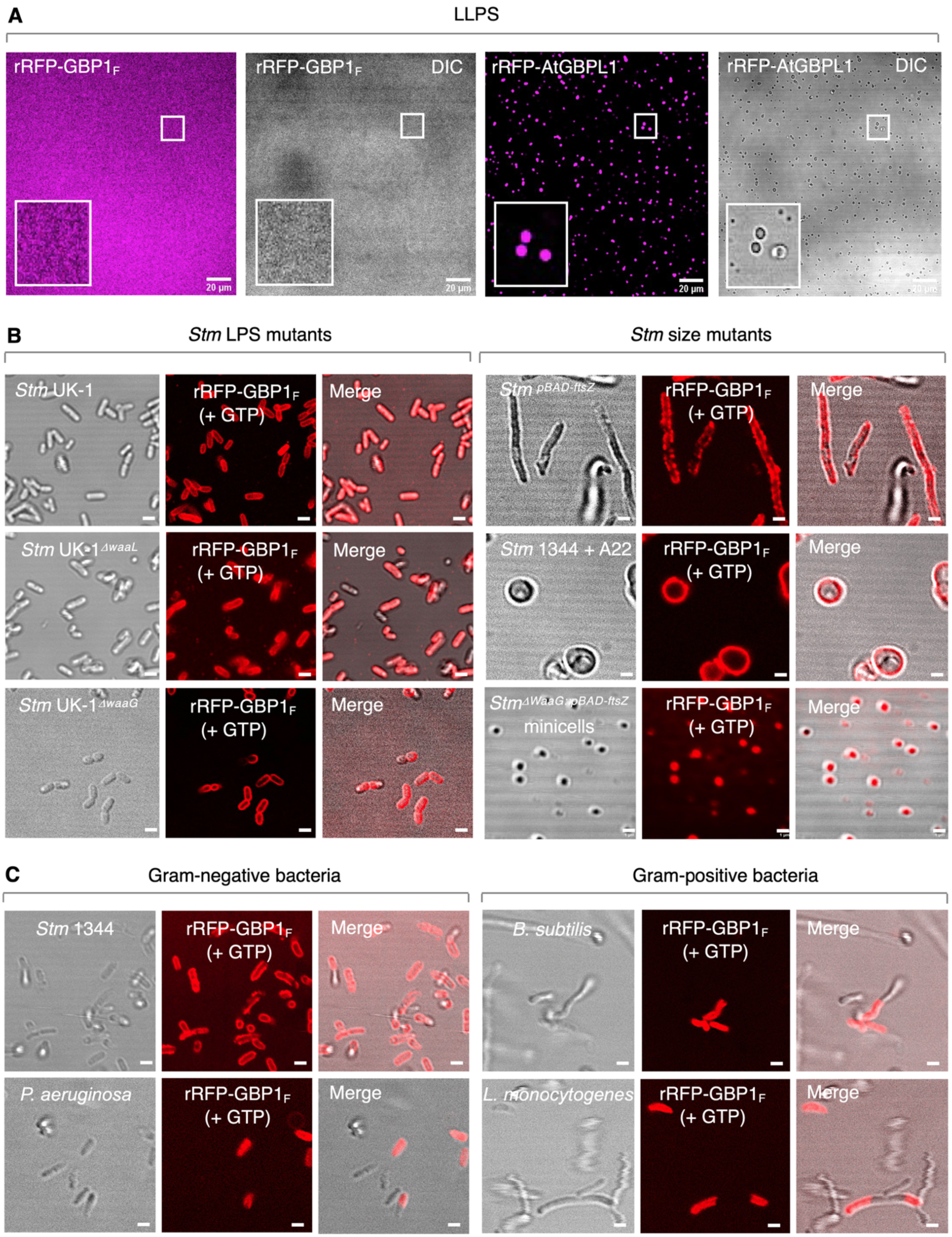
A
LLPS
rRFP-GBP1F
rRFP-GBP1F
DIC
rRFP-AtGBPL1
rRFP-AtGBPL1
DIC
20 µm
B
Stm LPS mutants
Stm size mutants
Stm UK-1
rRFP-GBP1F (+ GTP)
Merge
Stm pBAD-ftsZ
Stm UK-1 ΔwaaL
Stm 1344 + A22
Stm UK-1 ΔwaaG
Stm ΔWaaG::pBAD-ftsZ minicells
C
Gram-negative bacteria
Gram-positive bacteria
Stm 1344
B. subtilis
P. aeruginosa
L. monocytogenes

Fig. S5. Reconstituted GBP1 phase separation properties and GTP-dependent coat complex formation on different bacterial mutants and species. (A) Liquid-liquid phase separation (LLPS) droplet assay without Ficoll for rRFP-GBP1 and rRFP-AtGBPL1, the latter as a positive control. Pseudo-colored scanning confocal images collected with a 63×/1.40 oil immersion objective. Inset, coacervated droplets evident for rRFP-AtGBPL1 but not rRFP-GBP1. 1 of 2 representative experiments. **(B)** GTP-dependent rRFP-GBP1 coat reconstitution using *Stm* UK-1 LPS mutants (left) or size variants (right). The latter included genetically altered $Stm^{pBAD::ftsZ}$ elongated strains, A22-treated (S-(3,4-dichlorobenzyl)isothiourea; 500mM) circular strains, and purified $Stm^{\Delta waaG::pBAD\text{-}ftsZ}$ minicells. Scale bar, 1 μm. **(C)** GTP-dependent coat reconstitution on Gram-negative and Gram-positive bacterial strains. Scale bar, 1 μm. Micrographs from 3-6 experiments for **(B)** and **(C)**.

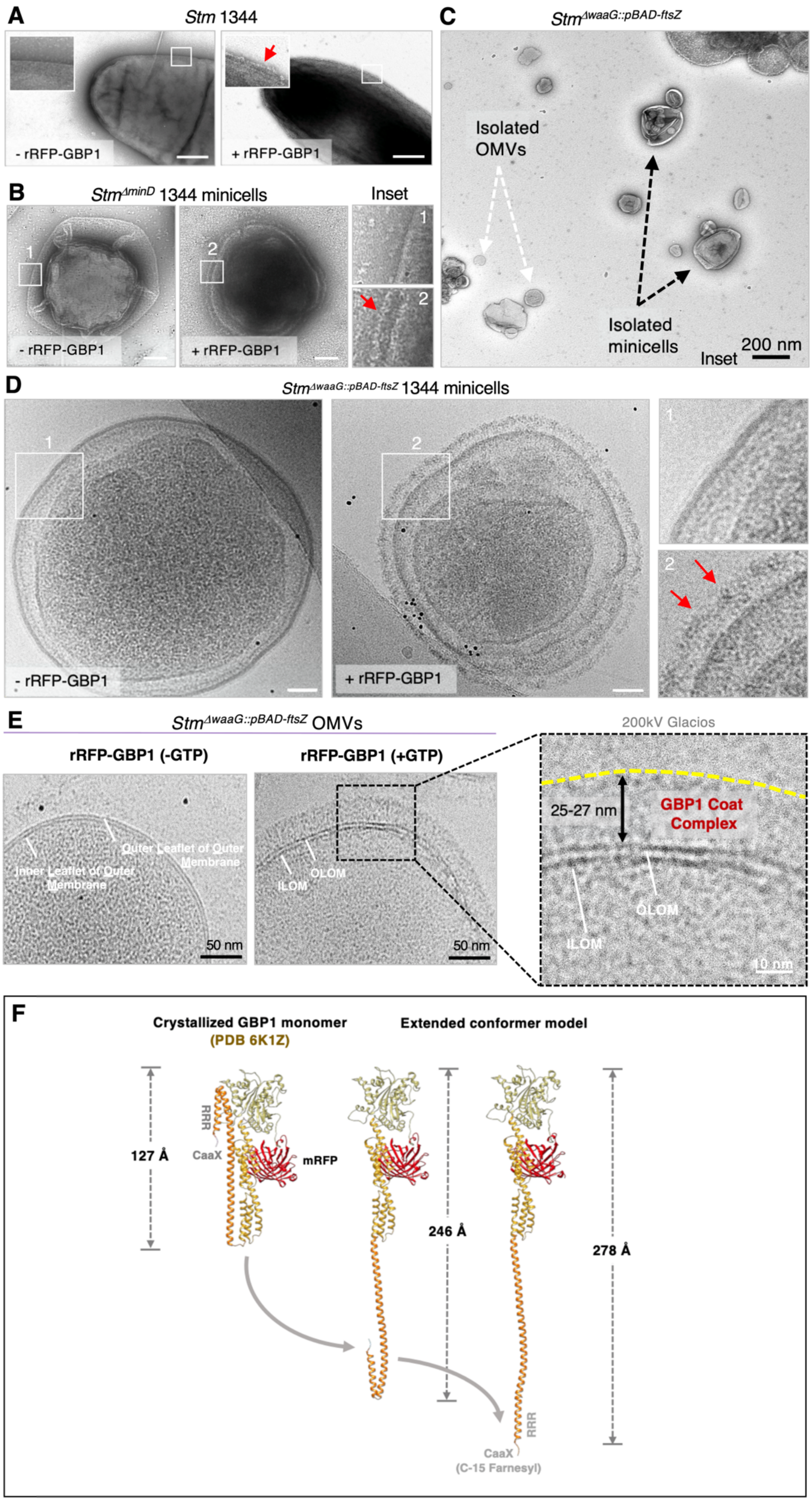
A
Stm 1344
- rRFP-GBP1
+ rRFP-GBP1
B
StmΔminD 1344 minicells
Inset
1
2
- rRFP-GBP1
+ rRFP-GBP1
C
StmΔwaaG::pBAD-ftsZ
Isolated OMVs
Isolated minicells
Inset
200 nm
D
StmΔwaaG::pBAD-ftsZ 1344 minicells
1
2
- rRFP-GBP1
+ rRFP-GBP1
E
StmΔwaaG::pBAD-ftsZ OMVs
200kV Glacios
rRFP-GBP1 (-GTP)
rRFP-GBP1 (+GTP)
Outer Leaflet of Outer Membrane
Inner Leaflet of Outer Membrane
ILOM
OLOM
50 nm
50 nm
25-27 nm
GBP1 Coat Complex
ILOM
OLOM
10 nm
F
Crystallized GBP1 monomer
(PDB 6K1Z)
Extended conformer model
127 Å
RRR
CaaX
mRFP
246 Å
278 Å
RRR
CaaX
(C-15 Farnesyl)

Fig. S6. *In vitro* reconstitution of GTP-dependent GBP1 coat assembly on *Salmonella* observed by negative stain EM and cryo-EM. (A) Negative stain EM of parental *Stm* 1344 incubated with rRFP-GBP1 in reconstitution assays. Inset, arrow demarcates GBP1 coat complex. Scale bar, 200 nm. (**B**) Negative stain EM of $Stm^{\Delta minD}$ minicells incubated with rRFP-GBP1 in reconstitution assays. Inset, arrow demarcates GBP1 coatomer. Scale bar, 50 nm. **(C)** Representative negative stain images showing isolated $Stm^{\Delta waaG::pBAD\text{-}ftsZ}$ fractions have both outer membrane vesicles (OMVs) and minicells. **(D)** Cryo-EM of isogenic $Stm^{\Delta waaG::pBAD\text{-}ftsZ}$ minicells incubated with rRFP-GBP1 in reconstitution assays. Inset, arrow demarcates GBP1 coat complex. 6 nm fuducial beads are present. Scale bar, 50 nm. (**E**) Estimated ~25-27 nm GBP1 coat length $Stm^{\Delta waaG::pBAD\text{-}ftsZ}$ OMVs in cryo-EM. OLOM, outer leaflet of outer membrane. ILOM, inner leaflet of outer membrane. (**F**) Dynamic GBP1 modeling based on PDB 6K1Z using initial size estimations from cryo-EM. Position of mRFP attached via a short amino acid linker to the N-terminus of GBP1 as modeled using AlphaFold2 (DeepMind) software. mRFP does not alter the length of the extended GBP1 conformer since it is constrained by a short amino acid linker.

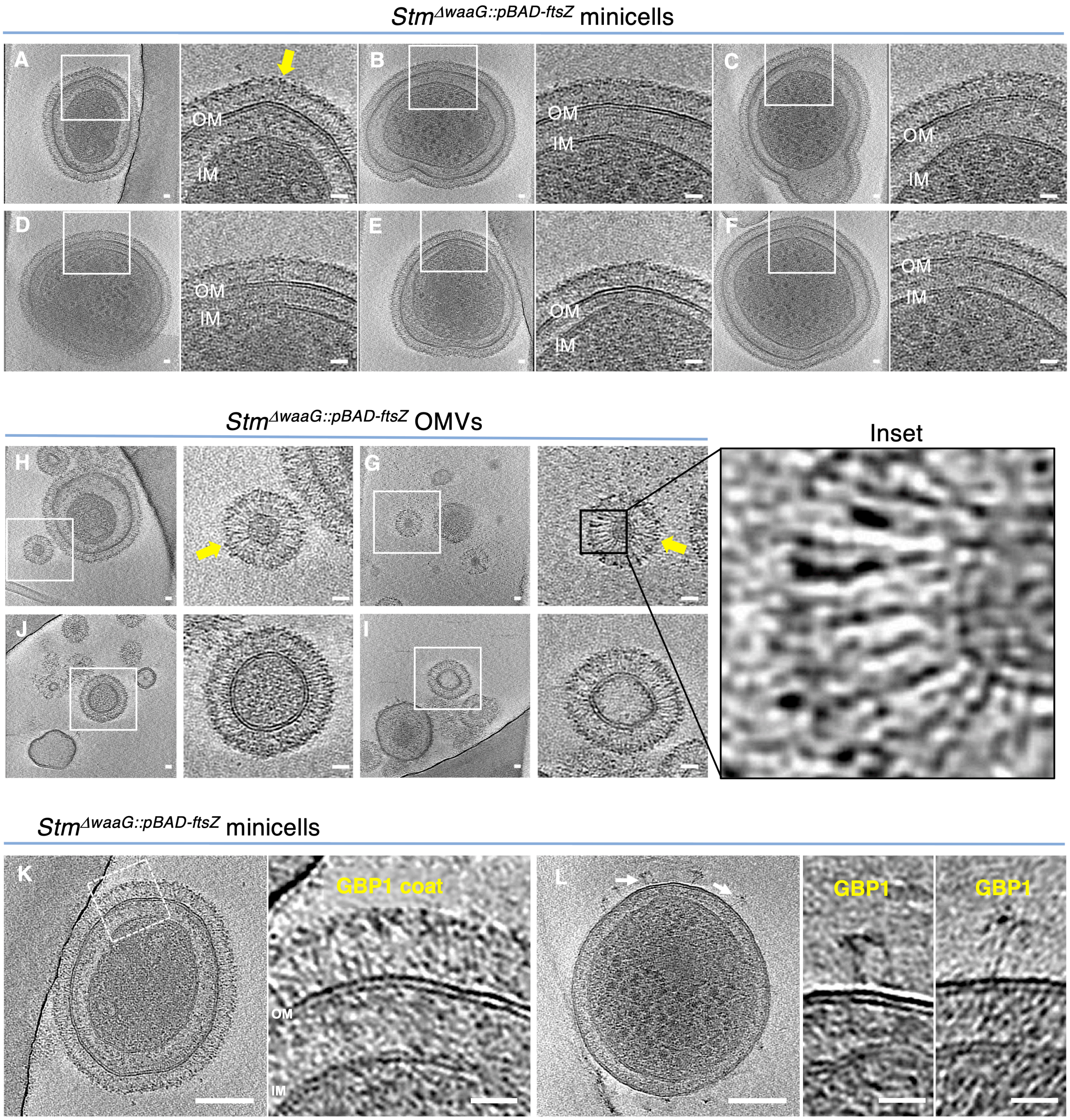

Fig. S7. 3D tomographic imaging of the GTP-dependent GBP1 coat complex. (A-J) 3D tomographic slices of different *Stm*$^{\Delta waaG::pBAD\text{-}ftsZ}$ minicells and OMVs coated in rRFP-GBP1. Yellow arrows highlight single elongated GBP1 conformers within insets. Scale bar, 20 nm. Inset in (G) depicts radiating "spoke" pattern of elongated GBP1 conformers in the native state. **(K,L)** *Stm*$^{\Delta waaG::pBAD\text{-}ftsZ}$ minicells showing sample before washing (K) and after washing (L) to remove protein crowdedness. White arrows, native GBP1 conformers. Scale bar, 50 nm. Insets, zoom-in view of boxed area **(K)** or GBP1 conformers **(L)**. Scale bar, 20 nm.

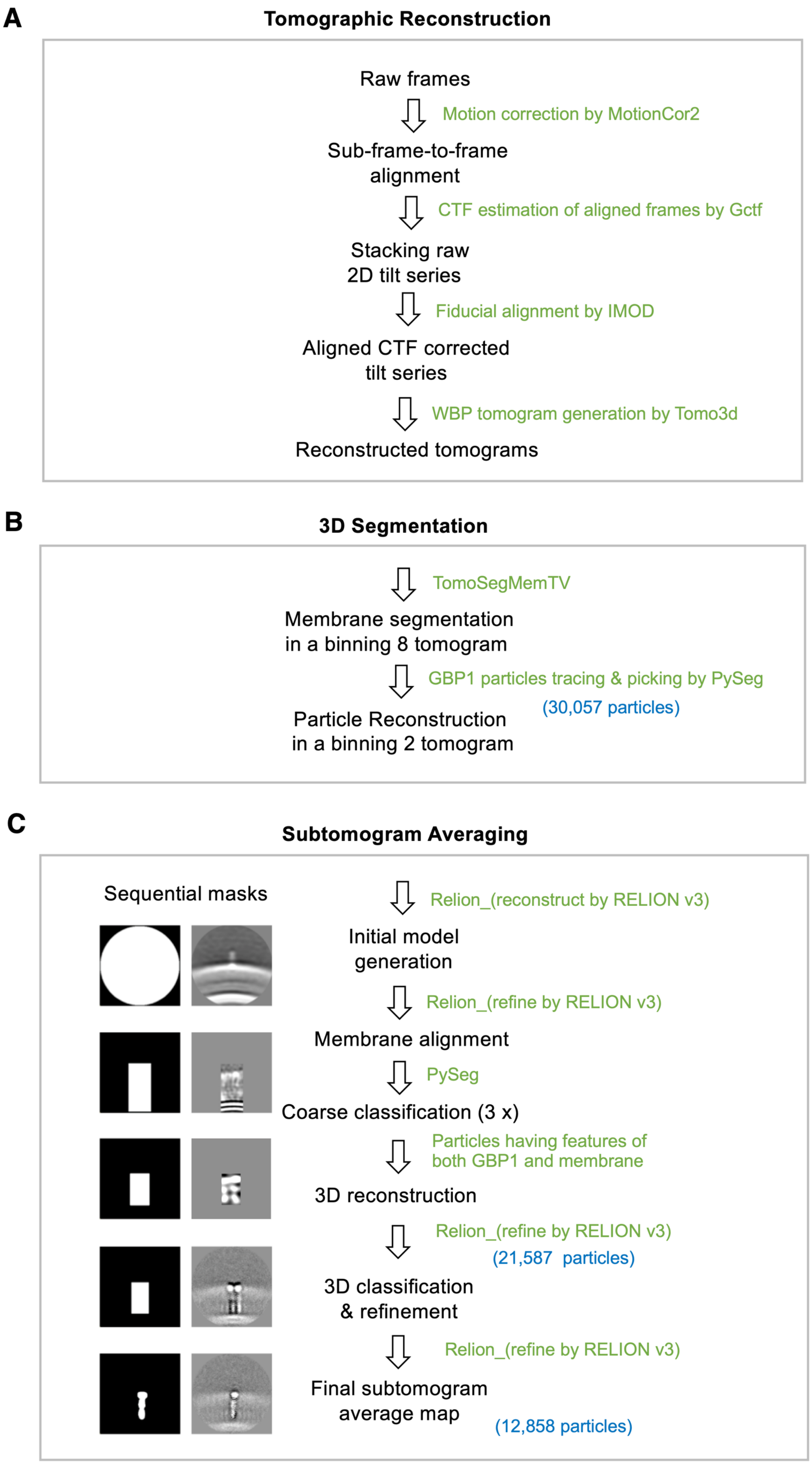
A
Tomographic Reconstruction
Raw frames
Motion correction by MotionCor2
Sub-frame-to-frame alignment
CTF estimation of aligned frames by Gctf
Stacking raw 2D tilt series
Fiducial alignment by IMOD
Aligned CTF corrected tilt series
WBP tomogram generation by Tomo3d
Reconstructed tomograms
B
3D Segmentation
TomoSegMemTV
Membrane segmentation in a binning 8 tomogram
GBP1 particles tracing & picking by PySeg
(30,057 particles)
Particle Reconstruction in a binning 2 tomogram
C
Subtomogram Averaging
Sequential masks
Relion_(reconstruct by RELION v3)
Initial model generation
Relion_(refine by RELION v3)
Membrane alignment
PySeg
Coarse classification (3 x)
Particles having features of both GBP1 and membrane
3D reconstruction
Relion_(refine by RELION v3)
(21,587 particles)
3D classification & refinement
Relion_(refine by RELION v3)
Final subtomogram average map
(12,858 particles)

Fig. S8. Workflow of 3D tomographic reconstruction for the untagged native GBP1 conformer. (A) Motion correction and alignment to generate weighted backprojection (WBP) tomograms for 3D segmentation. **(B)** Membrane segmentation via TomoSegMan TV and template-free particle tracing and picking enlisted the python package, PySeg. **(C)** Subtomographic averaging after coarse and refined classification for both thinner *Stm*$^{\Delta waaG}$ and thicker *Stm*$^{\Delta minD}$ particles. Number of particles collected at each step are indicated in blue font Last sequential mask shown is monomeric which yielded the highest final resolution (9.7 Angstroms).

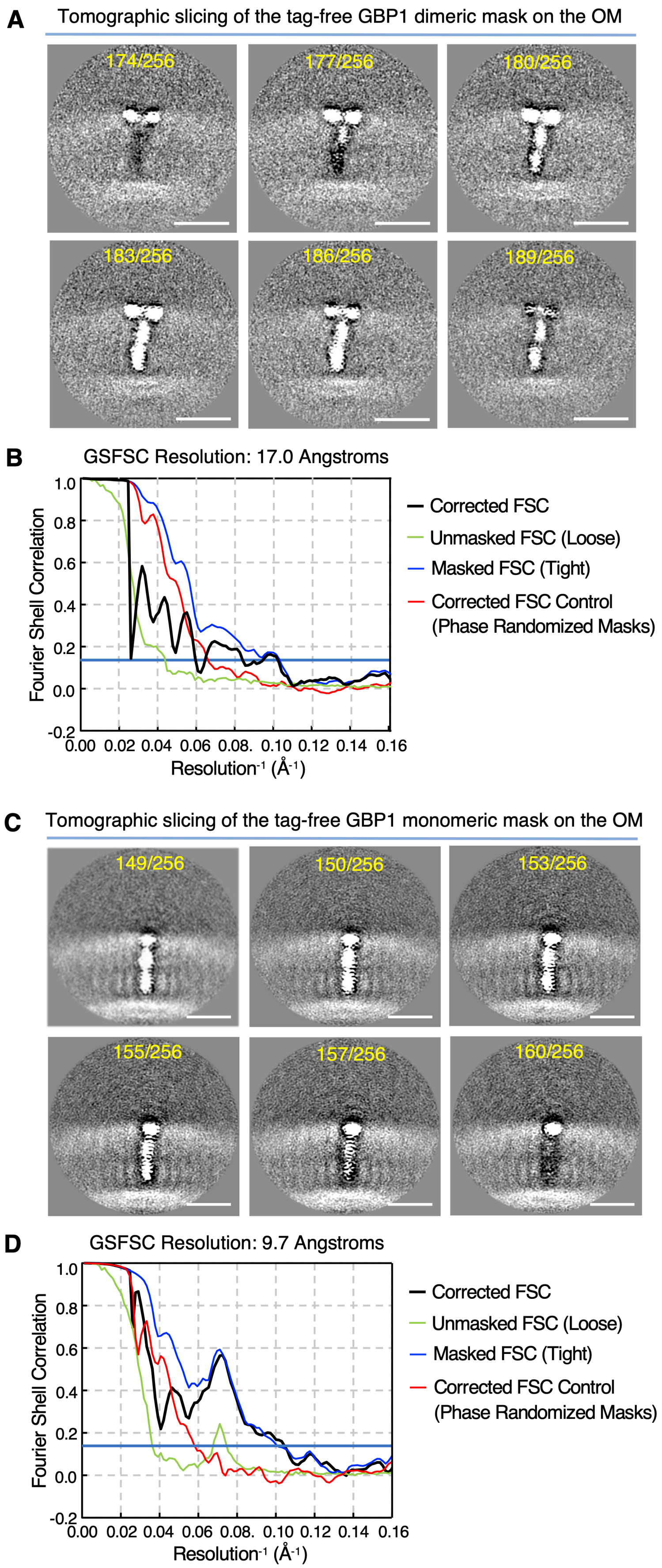
A
Tomographic slicing of the tag-free GBP1 dimeric mask on the OM
174/256
177/256
180/256
183/256
186/256
189/256
B
GSFSC Resolution: 17.0 Angstroms
Fourier Shell Correlation
Resolution-1 (Å-1)
Corrected FSC
Unmasked FSC (Loose)
Masked FSC (Tight)
Corrected FSC Control
(Phase Randomized Masks)
C
Tomographic slicing of the tag-free GBP1 monomeric mask on the OM
149/256
150/256
153/256
155/256
157/256
160/256
D
GSFSC Resolution: 9.7 Angstroms
Fourier Shell Correlation
Resolution-1 (Å-1)
Corrected FSC
Unmasked FSC (Loose)
Masked FSC (Tight)
Corrected FSC Control
(Phase Randomized Masks)

Fig. S9. Tomographic slicing through the 3D untagged GBP1 conformer directly on the bacterial OM. (A) Individual 2D tomographic slices through the 3D x,y plane volume of untagged GBP1 on *Stm*$^{\Delta waaG::pBAD\text{-}ftsZ}$ OMVs. Individual slices within 256*256 voxels using a larger mask on the intact dimer. **(B)** Fourier shell correlation reveals average coat complex resolution in Angstroms (black line) using a complete dimeric mask with appropriate controls. **(C)** Individual 2D tomographic slices through the 3D x,y plane volume using a smaller monomeric mask on the GBP1 dimer. Individual slices within 256*256 voxels. **(D)** Fourier shell correlation reveals average coat complex resolution in Angstroms (black line) derived from a monomeric mask with appropriate controls.

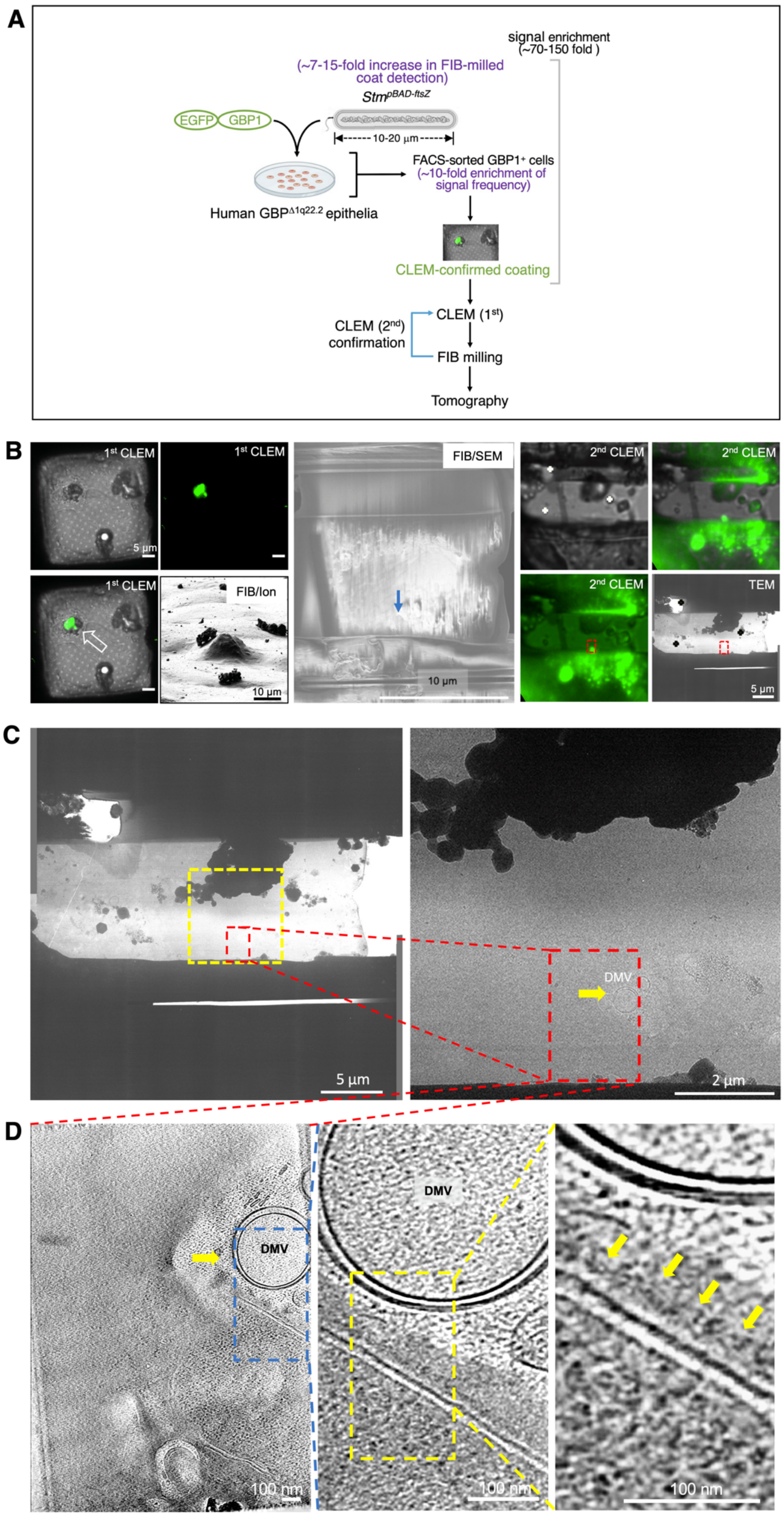
A
signal enrichment
(~70-150 fold)
(~7-15-fold increase in FIB-milled coat detection)
Stm^pBAD-ftsZ
EGFP
GBP1
10-20 μm
FACS-sorted GBP1+ cells
(~10-fold enrichment of signal frequency)
Human GBP^Δ1q22.2 epithelia
CLEM-confirmed coating
CLEM (1st)
CLEM (2nd) confirmation
FIB milling
Tomography
B
1st CLEM
5 μm
FIB/SEM
2nd CLEM
FIB/Ion
10 μm
TEM
C
DMV
5 μm
2 μm
D
DMV
100 nm

Fig. S10. *In situ* GBP1 coat detection by cryo-ET on FIB-milled and CLEM-verified lamellae. **(A)** Cryo-CLEM workflow of EGFP-GBP1 detection *in situ*. **(B)** 1st Cryo-CLEM, FIB and 2nd cryo-CLEM validation of EGFP-GBP1 coating on elongated $Stm^{pBAD\text{-}ftsZ}$ within $GBP^{\Delta 1q22.2}$ cells. Arrow, milling target for EGFP-GBP1 coat complex. 1st CLEM image was taken with DIC and GFP fluorescent channels. FIB/Ion, focused-ion beam image before rough milling; FIB/SEM, SEM image after fine milling; 2nd CLEM overlay, cryo-CLEM observation confirming EGFP-hGBP1 fluorescent signal in the grid after fine milling of FIB; TEM-Montage; transmission EM of the milled lamellae. Dashed rectangle, area where cryo-ET tilt series were collected and matched to the top panel of tomogram in **(C)**. Crosses denote ice deposits also used for positional overlay. **(C,D)** Positioning of the collected tomographic area within the larger lamellae. Single yellow area denotes the double-membrane vesicle (DMV) for helping locate coated bacilli and multiple yellow arrows indicate the putative GBP1 coat itself within the inset. Scale bar, 100 nm.

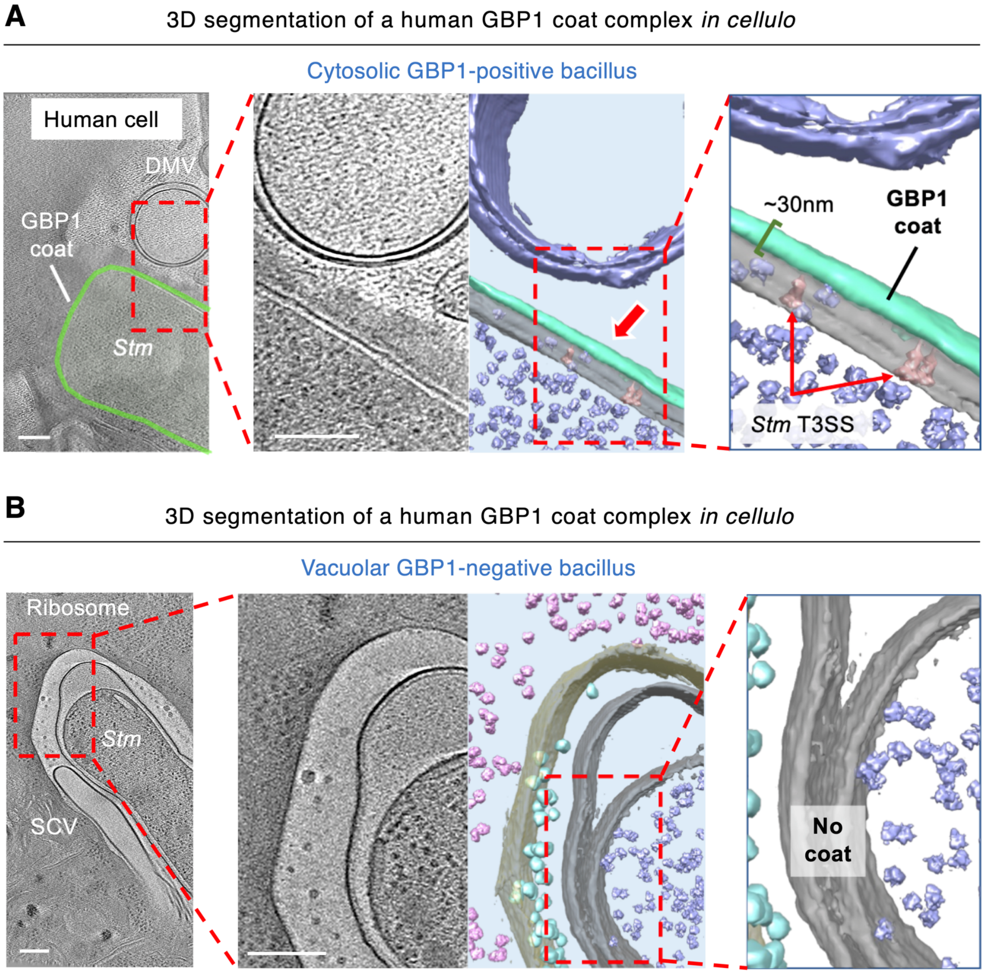

Fig. S11. *In situ* GBP1 coat visualization by cryo-ET on FIB-milled and CLEM-verified lamellae.

(A) *In situ* tomographic slices with 17.2 nm thickness. Cytosolic $Stm^{pBAD\text{-}ftsZ}$ was targeted by EGFP-GBP1 reconstituted in $GBP^{\Delta 1q22.2}$ cells. DMV, double-membrane vesicle. Zoomed-in area at right shows GBP1 coat complex (red arrow) and bacterial type 3 secretion system (T3SS) complex. Scale bar, 200 nm. **(B)** Representative tomographic slice of vacuolar $Stm^{pBAD\text{-}ftsZ}$ lacking GBP1 coating. Inset, size of GBP1 coat and underlying T3SS. In these 3D segmented images, cytosolic free $GBP^{\Delta 1q22.2}$ cell ribosomes are indicated in the pink; Bacterial ribosomes are segmented in the purple. SCV, *Salmonella*-containing vacuole. Scale bar, 200 nm.

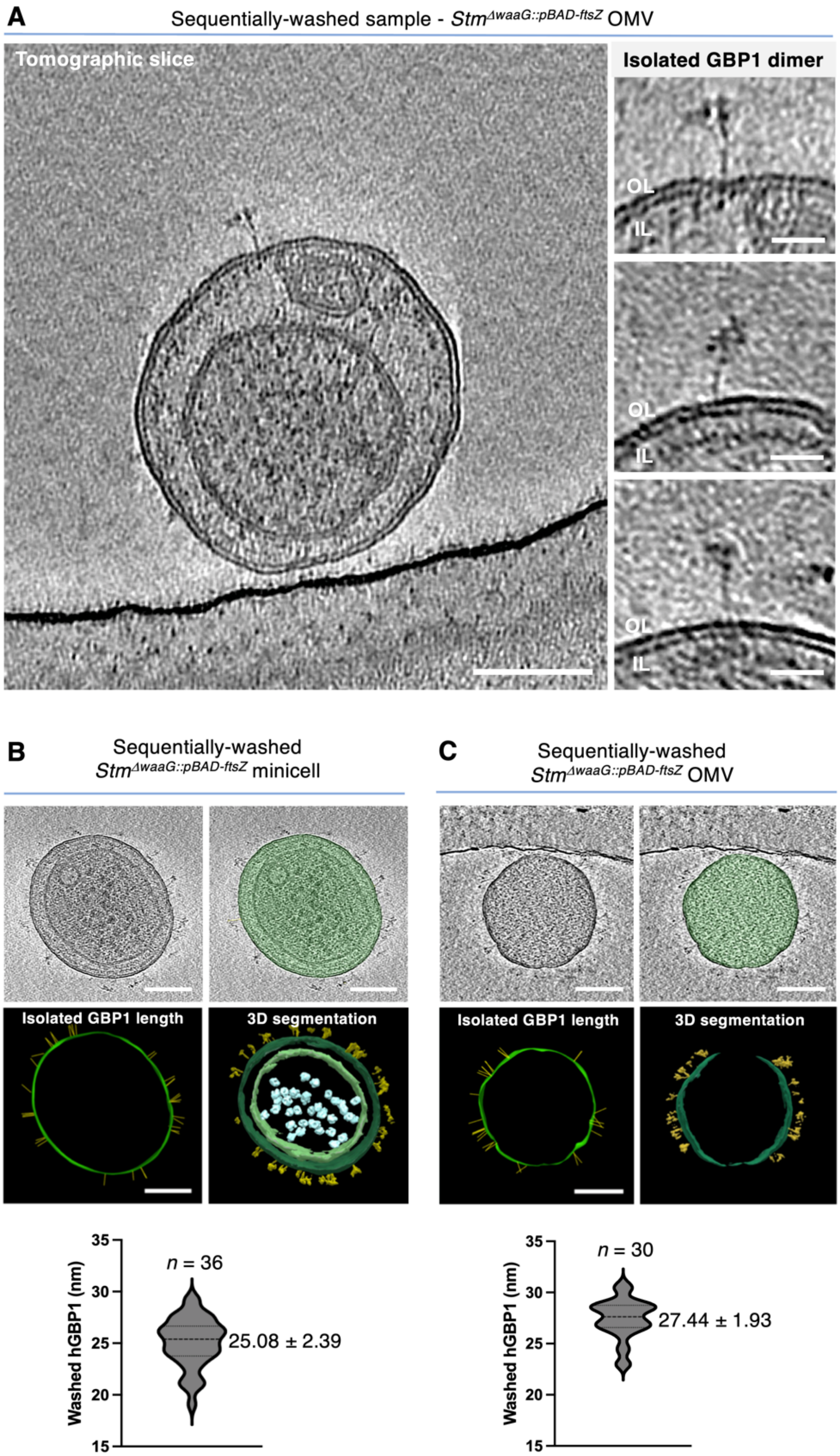
A
Sequentially-washed sample - Stm^ΔwaaG::pBAD-ftsZ OMV
Tomographic slice
Isolated GBP1 dimer
OL
IL
OL
IL
OL
IL
B
Sequentially-washed
Stm^ΔwaaG::pBAD-ftsZ minicell
Isolated GBP1 length
3D segmentation
Washed hGBP1 (nm)
35
30
25
20
15
n = 36
25.08 ± 2.39
C
Sequentially-washed
Stm^ΔwaaG::pBAD-ftsZ OMV
Isolated GBP1 length
3D segmentation
Washed hGBP1 (nm)
35
30
25
20
15
n = 30
27.44 ± 1.93

Fig. S12. Upright GBP1 conformers with C-terminal attachment shown in sequentially washed samples. (A) (Left) Tomographic slice of a sequentially washed *Stm*$^{\Delta waaG::pBAD\text{-}ftsZ}$ OMV. (Right) Examples of isolated GBP1 dimers identified in sequentially washed samples with the globular GD at the top and extended C-terminal tail underneath. **(B)** 3D segmentation and length of isolated GBP1 conformers on sequentially washed *Stm*$^{\Delta waaG::pBAD\text{-}ftsZ}$ minicells. (**C**). 3D segmentation and length of isolated GBP1 conformers on sequentially washed *Stm*$^{\Delta waaG::pBAD\text{-}ftsZ}$ OMVs. 1 of 3 independent experiments.

A

Human GBP1 cysteine replacement strategy

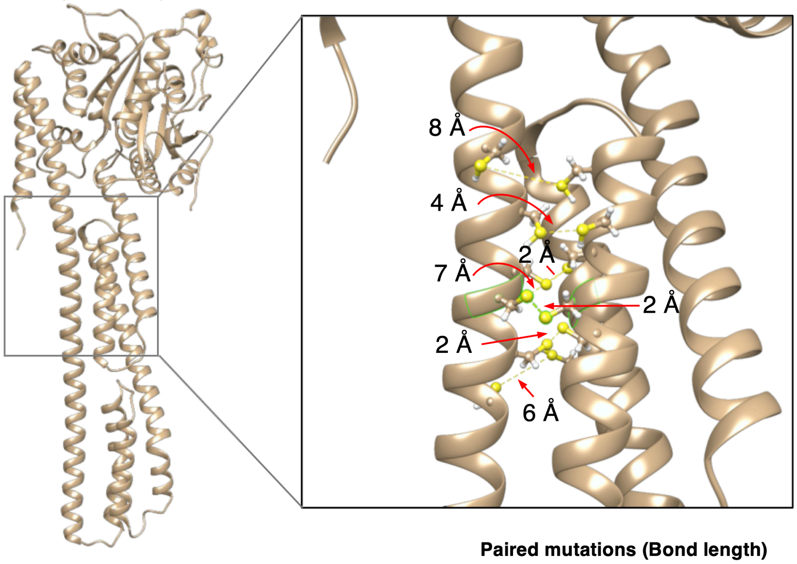

Single mutations

Mutations (Helix 7)	Mutations (Helix 12)
Gln351Cys	Met516Cys
Leu354Cys	Glu518Cys
Arg358Cys	Gln519Cys
Glu361Cys	Lys520Cys
Arg362Cys	Arg522Cys
Ile365Cys	Ser523Cys
Glu366Cys	Glu526Cys
Ile369Cys	His527Cys
Glu389Cys	Gln530Cys
Arg392Cys	Glu533Cys

Paired mutations (Bond length)

1. Ile369Cys-Glu533Cys (8 Å)
2. Glu366Cys-Gln530Cys (4 Å)
3. Ile365Cys-His527Cys (2 Å)
4. **Arg362Cys-Glu526Cys** (2 Å)
5. Ile365Cys-Glu526Cys (7 Å)
6. Glu361Cys-Ser523Cys (2 Å)
7. Glu389Cys-Lys520Cys (6 Å)

B

GBP$^{\Delta chr.1q22.2}$ + human GBP1 Cys mutants (SIM imaging)

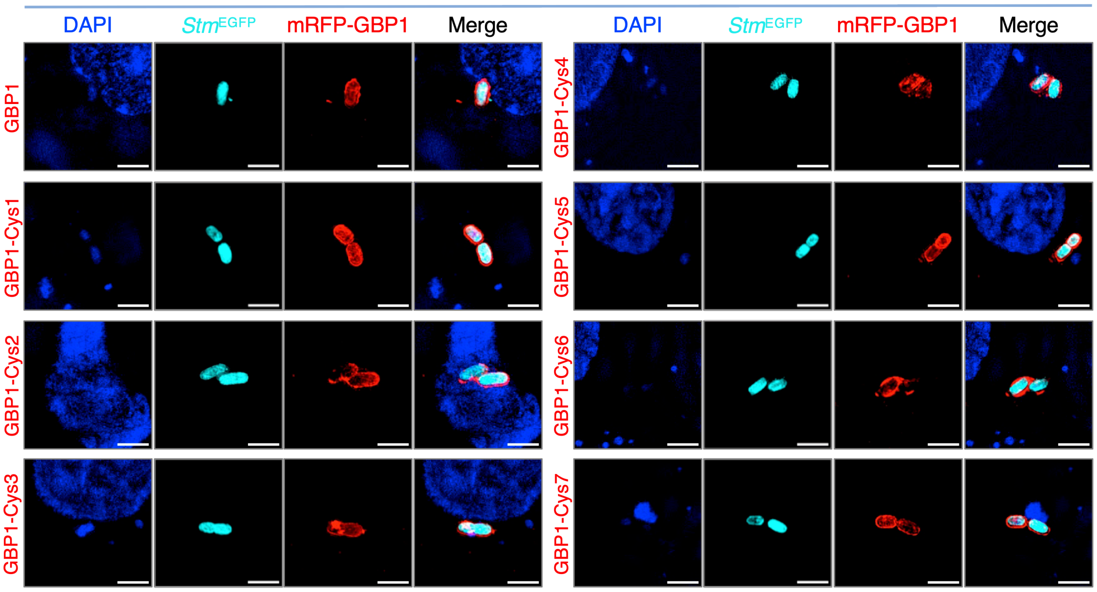

Fig. S13. Cysteine replacement strategy for open versus closed GBP1 conformer requirement in building the coat complex. (A) Position of amino acid substitutions in the α7 and α12 helices to generate paired disulfide linkages and their respective bond lengths. The Arg352Cys-Glu526Cys pair used for dynamic coating analysis shown in bold type. **(B)** SIM imaging of the *Stm* coat generated by all transfected Cys-Cy mutant pairs versus wild-type GBP1 under the reducing environment of the HeLa cell cytosol. It confirms no gross structural changes were introduced that might interfere with cell-free assembly. Scale bar, 2μm. 1 of 2 similar experiments shown. GFP-expressing *Stm* has been pseudocolored.

Table S1. Antibodies used in this study

IB: immunoblot, IF: immunofluorescence

Antigen	Species IgG/IgM	Application (Dilution)	Source & catalog number
Actin (AC-15)	Mouse	IB (1:5000)	Abcam; Ab6276
Caspase-4 (4B9)	Mouse	IB (1:1000)	Enzo; ADI-AAM-114-E
Donkey anti-Goat IgG-HRP	Goat	IB (1:1000)	ThermoFisher; PA1-28664
DsRed	Mouse	IB (1:5000)	Santa Cruz Biotechnology; sc-390909
FLAG (M2)	Mouse	IB (1:10000), IF (1:1000)	Sigma-Aldrich; F1804
Fli-C	Mouse	IF (1:1000)	BioLegend; 629702
GAPDH	Rabbit	IB (1:5000)	Santa Cruz Biotechnology; sc-25778
GBP1	Rat	IB (1:2000), IF (1:300)	Santa Cruz Biotechnology; sc-53857
GBP2	Mouse	IB (1:200)<br>IB (1:200)	Origene/Santa Cruz Biotechnology; TA500657/sc-10581
GFP	Mouse	IB (1:5000)	Roche/SigmaGenescript; 11814460001/ A01704
Goat anti-Rat IgG (H+L)-HRP	Rat	IB (1:1000)	ThermoFisher; 31470
GSDMD	Rabbit	IB (1:1000)	Novus Biologicals; NBP2-33422
HA.11 (16B12)	Mouse	IB (1:10000), IF (1:1000)	BioLegend; 901501
IgG (Goat)-Alexa488or 594 or 647	Donkey	IF (1:1000)	ThermoFisher; A11055
IgG (Mouse)-Alexa488 or 594 or 647	Donkey	IF (1:1000)	ThermoFisher; A21203

IgG (Rabbit)-Alexa488 or 594 or 647	Donkey	IF (1:1000)	ThermoFisher; A21207
Interleukin-18	Rabbit	IB (1:1000)	MBL International; PM014
Mouse IgG, HRP-linked whole Ab	Mouse	IB (1:1000)	GE Healthcare; NXA931-1ML
Rabbit IgG, HRP-linked whole Ab (from donkey)	Rabbit	IB (1:1000)	GE Healthcare; NA934-1ML
Salmonella O-antigen Group B antisera	Rabbit	IF (1:20000)	BD Biosciences; 240984
Streptavidin, Alexa Fluor® 647 Conjugate		IF (1:1000)	ThermoFisher; S32357

Table S2. Bacterial strains used in this study
BG, Background

Strain	Isogenic BG	Plasmid backbone	Insertion/ Mutation	Antibiotic Resistance	Origin
Stm$^{\Delta minD}$	1344	N/A	minD deletion/Km insertion	Kanamycin	This study
Stm$^{mreB(K27E)}$	1344	N/A	mreB (K27E)		This study
Stm$^{mreB(D78V)}$	1344	N/A	minD deletion/Km insertion	Kanamycin	This study
Stm$^{Scarlet\text{-}I}$	1344	pmScarlet- i_C1		Kanamycin	This study
Stm^{EGFP}	1344	pEGFP			This study
strain 1344		N/A			J.E. Galan
Stm$^{\Delta flhD}$	1344	N/A	flhD deletion		J.E. Galan
Stm UK-1		N/A			R. Curtis III
Stm$^{pBAD::ftsZ}$	UK-1	pBAD24::ftsZ		Ampicillin	This study
Stm$^{\Delta waaG::pBAD\text{-}ftsZ}$	UK-1	pBAD24::ftsZ	waaG deletion	Ampicillin	This study
Stm$^{\Delta wzy}$	UK-1	N/A	wzy deletion		R. Curtis III
Stm$^{\Delta waaL}$	UK-1	N/A	waaL deletion		R. Curtis III
Stm$^{\Delta waaJ}$	UK-1	N/A	waaJ deletion		R. Curtis III
Stm$^{\Delta waaI}$	UK-1	N/A	waaI deletion		R. Curtis III
Stm$^{\Delta waaG}$	UK-1	N/A	waaG deletion		R. Curtis III
Stm$^{\Delta waaG}$	UK-1	N/A	waaG deletion		R. Curtis III
Stm$^{\Delta lpxR}$	UK-1	N/A	lpxR deletion		R. Curtis III
Stm$^{\Delta pagL}$	UK-1	N/A	pagL deletion		R. Curtis III
Stm$^{\Delta pagP}$	UK-1	N/A	pagP deletion		R. Curtis III
Stm$^{\Delta lpxR \Delta pagL \Delta pagP}$ (χ11088)	UK-1	N/A	lpxR, pagL, pagP triple deletion		R. Curtis III

Table S3. Guide sequences and genomic PCR primers for CRISPR-Cas9 gene targeting

**Loci**	**sgRNA or genomic primer**	**5' side**	**3' side**
AOAH	sgRNA	**Sense**<br>CCATTTGAGGCTACGCACTG	**Sense**<br>CGGTGAAACAGAAGAGA TGGGTAT
	genomic primers	**Forward**<br>CCATTTGAGGCTACGCACTG	**Reverse**<br>CGGTGAAACAGAAGAGA TGGGTAT
CASP4	sgRNA	**Antisense**<br>TACGTTGCTTCTCTTGCATA	**Sense**<br>GAGAAACAACCGCACACGCC
	genomic primers	**Forward**<br>AGAGGAGCTGCATGAAGA CT	**Reverse**<br>ATGTATGTGTTTGTGGCG GC
GBPchr. 1q22.2	sgRNA	**Antisense on GBP6 exon 3**<br>AAGGTCTGGGCGATGTGG AA	**Antisense on GBP3 exon 4**<br>TGTACACGAGAGTGCTGC TC
	genomic primers	**Forward**<br>CTCTTTGTGGGCAGGACCT TTACTTT	**Reverse**<br>AGCTGAGTAGCTAACTAA GGAAATGTGA
GBP1	sgRNA	**Antisense**<br>TTTAGTGTGAGACTGCACCG	**Sense**<br>GTGCCCCACCCCAAGAAG CC
	KO detection genomic primers	**Forward**<br>CGTAAGGTGAATACTGATT GTTGT	**Reverse**<br>ACCCACACATATTACAGC CTGT
GBP2	sgRNA	**Sense**<br>CCTAGTTCTGCTCGACACT G	**Antisense**<br>CCTCAGTGTCGAGCAGAA CT

	genomic primers	**Forward**<br><br>TCTCAATTTCCAGAAACAC CTACTAAG	**Reverse**<br><br>TGTACTCTGAATGCTTAT ACCACACA
GBP3	sgRNA	**Sense**<br><br>TACCTGATGAACAAGCTA GC	**Antisense**<br><br>TGTACACGAGAGTGCTGC TC
	genomic primers	**Forward**<br><br>TGCCAATGATCATAAGAG GACCT	**Reverse**<br><br>TGTGACAAAATGAACAGG AACCT
GBP4	sgRNA	**Sense**<br><br>ATTGTAGGGCTATACCGCA C	**Sense**<br><br>TATCTCATGAATCGTCTTG C
	genomic primers	**Forward**<br><br>TGTGCTTTTCTCCGAGTCA G	**Reverse**<br><br>CCCTTTCCTCCTCCAACTT CT
GSDMD	sgRNA	**Sense**<br><br>CTTGCTTTAGACGTGCAGCG	**antisense**<br><br>CGCTGCACGTCTAAAGCAAG
	genomic primers	**Forward**<br><br>GAGCCCCAGGGAGGCTGGAT	**Reverse**<br><br>GCAGGGTTGCTTGGGGTAGG
RNF213	sgRNA	**Sense**<br><br>TGACTTTGCTTTCAAACCCG	**Antisense**<br><br>CGGGTTTGAAAGCAAAGTCA
	genomic primers	**Forward**<br><br>GGAACAAGTCCGCTTCCTCA	**Reverse**<br><br>AAATGAAAGAGGCGCAGCCG

Table S4. Summary of GBP1 mutant phenotypes in Fig. 1D-H, 3F and fig. S2B-D.

**GBP Mutant**	**Catalytic activity (GTPase; GDP produced)**	**Catalytic activity (GDPase; GMP produced)**	**Self-assembly**	**C-15 farnesyl group**	**LPS binding**	***Stm* targeting & lipid A release (cell-free)**	***Stm* targeting, pyroptosis and IL8 secretion (*in situ*)**
GBP1	Y	Y	Y	Y	Y	Y	Y
$GBP1^{S52N}$	N	N	N	Y	N	N	N
$GBP1^{DD103.108NN}$	Y	N	N	Y	N	N	N
$GBP1^{D184N}$	Y	Y	N	Y	N	N	N
$GBP1^{C589S}$	Y	Y	Y	N	N	N	N
$GBP1^{R584-586A}$	Y	Y	Y	Y	N	N	N

Movie S1. 3D-SIM imaging of cytosolic GBP1 coat assembly in *Stm*-infected human cells. GBP1$^{-/-}$ HeLa cells expressing RFP-GBP1 and infected with EGFP-expressing *Salmonella*. OMX-SR images are maximum projections from 5µm stacks and visualized by Imaris software.

Movie S2. 3D-SIM imaging of cytosolic GBP1 coat assembly in *Stm*-infected human cells. GBP1$^{-/-}$ HeLa cells expressing RFP-GBP1 and infected with EGFP-expressing *Salmonella*. OMX-SR images are maximum projections from 5µm stacks without post-acquisition Imaris analysis.

Movie S3. 4Pi-SMS nanoscopic imaging of GBPs on *Salmonella* in IFN-γ-activated human epithelial cells. 360° rotation of endogenous human GBP1 and GBP2 detected by antibody staining on *Stm* 1344 within the cytosol of in IFN-γ-activated HeLa cells. Images acquired at 60 min post-infection. Removing each label reveals endogenous GBP1 has completely coated bacteria underneath native GBP2.

Movie S4. GBP1 conformer length measurements of *Stm*$^{\Delta minD}$ minicell. Example of computer script programs measuring coat lengths between outer and inner perimeters of minicells that were first assigned manually to ensure correct assignment in different raw tomographic slices.

Movie S5. GBP1 conformer length measurements of *Stm*$^{\Delta waaG::pBAD\text{-}ftsZ}$ minicell. Example of computer script programs measuring coat lengths between outer and inner perimeters of minicells that were first assigned manually to ensure correct assignment in different raw tomographic slices.

Movie S6. 3D segmentation of the GBP1 coat complex on a *Stm*$^{\Delta waaG::pBAD\text{-}ftsZ}$ minicell. Overlay of 3D segmentation onto raw tomographic slice from which subtomographic averaging was derived. Positions of the GBP1 coat, OM, IM and ribosomes are shown.
